# Uptake of Micro‐ and Nano‐Sized Plastic Particles in Human Cells: Size‐Dependent Endocytosis and Cellular Responses

**DOI:** 10.1155/bmri/4933959

**Published:** 2026-10-21

**Authors:** Alisha M. Janiga-MacNelly, Ramon Lavado

**Affiliations:** ^1^ School of Earth and Environmental Sciences Baylor University Waco Texas USA baylor.edu

**Keywords:** cellular uptake, endocytosis, human cell models, in vitro, nanoplastics

## Abstract

Micro‐ and nanoplastics (MNPs) are increasingly detected in human tissues, yet the mechanisms governing their cellular uptake remain poorly understood. This study evaluated size‐dependent cellular association of polystyrene MNPs (0.25–1.12 *μ*m) across seven human cell lines representing skin, intestine, lung, kidney, liver, brain, and vascular endothelium. No cytotoxicity was observed in six cell lines after 24 or 48 h at concentrations up to 100 *μ*g/mL, and epithelial and endothelial barrier integrity remained unaffected. Several cell types showed increased metabolic activity after 48 h. Cell‐associated MNP fluorescence exhibited cell‐type‐ and particle‐size‐dependent patterns. HEK001 keratinocytes and HMC‐3 microglia showed rapid increases in cell‐associated particle signals, with an apparent plateau for 0.25 *μ*m particles; whereas HEK293‐hTLR2 kidney cells and MRC‐5 lung fibroblasts exhibited increasing cell‐associated fluorescence over time across several particle sizes. Caco‐2 intestinal cells showed limited interaction, whereas HepaRG liver and HMEC‐1 endothelial cells exhibited size‐dependent responses. Inhibition studies indicated that actin‐dependent pathways contributed predominantly to the association of 0.54–1.12 *μ*m particles with cells, whereas clathrin‐mediated endocytosis contributed to smaller‐particle interactions in a highly cell‐specific and size‐specific manner. Polydisperse mixtures exhibited reduced cell‐associated particle signals but were unaffected by endocytosis inhibitors, further highlighting the complexity of nanoplastic internalization and the importance of both tissue identity and particle size in determining cellular interactions.

## 1. Introduction

Plastic production has increased exponentially in recent decades, reaching approximately 400 Mt globally in 2022, with more than 200 Mt entering waste streams [1]. Environmental degradation of plastic waste produces micro‐ and nanoplastics (MNPs), defined as particles ranging from 1 *μ*m to 1 mm (microplastics, MPs) and 1 nm to 1 *μ*m (nanoplastics,NPs) [2]. These particles are now pervasive across environmental matrices, with concentrations reported up to 800 particles/m^3^ in marine systems [3], 10 Tg/year in the atmosphere [4], and over 1000 MPs/kg in soils [5].

Human exposure to MNPs is now well documented. Particles have been detected in multiple tissues, including colon, lung, liver, kidney, brain, and blood, spanning a wide size range from nanometers to millimeters [6]. Although ingestion remains a primary exposure route through contaminated food and packaging, inhalation and dermal exposure also contribute, particularly under occupational or urban conditions [7, 8]. Additionally, emerging evidence highlights medical sources of exposure, such as airborne MPs in surgical environments and leaching from medical devices [9, 10]. Despite this widespread exposure, the mechanisms by which MNPs interact with and enter human cells remain poorly characterized.

A key limitation in the current literature is the uneven representation of tissues and cell types. For example, the skin has been relatively understudied because researchers assumed the epidermal barrier prevented particle penetration. However, experimental evidence demonstrates that NPs (~100 nm) can penetrate all epidermal layers when the stratum corneum is compromised [11], and keratinocyte uptake has been observed for particles up to 500 nm [11, 12]. Importantly, basal keratinocytes, critical for wound healing, immune signaling, and angiogenesis, have not been investigated, despite their increased exposure under damaged skin conditions [13]. This gap is addressed here using the HEK001 cell line as a representative model.

Similarly, gastrointestinal exposure has been extensively modeled using Caco‐2 cells; however, most studies have focused on particles ≤ 100 nm, which does not reflect the larger particle sizes reported in human colon tissues [6, 14–17]. Expanding the size range of tested particles is therefore necessary to better approximate realistic exposure scenarios.

Inhalation represents another significant exposure pathway. NPs can reach the alveolar region, with smaller particles capable of translocating into systemic circulation [18, 19]. Lung fibroblasts, located between epithelial and endothelial barriers, play essential roles in extracellular matrix maintenance, inflammation, and tissue repair [20]. Although previous work demonstrated size‐dependent cytotoxicity in fibroblasts [21], the mechanisms governing NP uptake remain unresolved. This study addresses this gap using MRC‐5 cells and extends exposure duration to capture more physiologically relevant dynamics.

Evidence of MNP accumulation in kidney and liver tissues further underscores the systemic distribution of these particles [22, 23]. Although in vitro studies have reported cellular interactions in renal and hepatic models [24–28], mechanistic insights remain limited. The present study utilizes HEK293‐hTLR2 cells to incorporate immune‐relevant signaling pathways [29–31], and HepaRG cells, which provide improved physiological relevance compared with commonly used hepatic models [32].

The potential for MNPs to affect the central nervous system is of particular concern. Although the blood‐brain barrier (BBB) is thought to restrict particles > 100 nm [33], evidence suggests that larger NPs may disrupt BBB integrity or enter via alternative routes such as the olfactory pathway [34–36]. Microglia, the resident immune cells of the brain, exhibit enhanced NP accumulation compared with other neural cell types [37, 38], yet their uptake mechanisms remain unclear. This study employs HMC‐3 cells to address this knowledge gap.

The detection of MNPs in blood [39–41] further suggests that particles must cross endothelial barriers. Endothelial cells regulate vascular permeability and transport processes [42–44], yet mechanistic understanding of MNP uptake remains incomplete despite evidence of internalization [45, 46]. HMEC‐1 cells were selected to model capillary endothelium, providing a more physiologically relevant system than commonly used umbilical vein models.

Across these systems, several critical gaps persist. Most studies focus on small NPs (≤ 100 nm), despite evidence that larger particles are prevalent in human tissues. Additionally, studies are often limited to short exposure durations (4–24 h) and a narrow range of cell types, despite the highly cell‐specific nature of endocytosis [3, 7, 47, 48]. Furthermore, many investigations assess toxicity without confirming particle uptake [6]. These limitations hinder the development of a comprehensive understanding of MNP behavior in human systems.

To address critical gaps in our understanding of MNP interactions with human systems, this study was designed to provide a comparative and mechanistic evaluation of MNP uptake across multiple tissues. We investigated size‐dependent internalization using an expanded particle‐size range (0.25–1.12 *μ*m) that overlaps with the sizes of MNPs reported in human tissues [6, 14]. Based on the size definition used here, particles ≤ 1 *μ*m are referred to as NPs, whereas the 1.12 *μ*m particles fall within the lower microplastic size range. In parallel, we employ a diverse panel of human cell lines representing key exposure and accumulation sites, including skin, gastrointestinal tract, lung, kidney, liver, brain, and vasculature, to capture the well‐established cell‐type specificity of endocytic processes [7, 47]. This comparative, multi‐tissue approach complements previous studies conducted in individual human cell models by enabling direct comparison of MNP responses across seven cell types under standardized experimental conditions.

In addition, this study incorporates short‐term exposures of up to 48 h to evaluate particle‐cell interaction dynamics over time and extend beyond the shorter exposure periods (≤ 24 h) commonly reported in the literature [8, 15]. Mechanistically, MNP internalization is questioned using pharmacological inhibitors in combination with imaging approaches to identify specific endocytic pathways, addressing the common limitation of assessing toxicity without confirming uptake [6]. Previous studies have established that polystyrene MNP uptake and endocytic mechanisms are influenced by particle size and cellular context. However, much of this evidence has been generated using individual or limited numbers of cell models, different particle characteristics, exposure conditions, and experimental approaches, making direct comparisons across tissues difficult. To address this limitation, the present study applies a common experimental framework to seven human cell models representing the skin, intestine, lung, kidney, liver, brain, and vascular endothelium. Using the same MNP sizes, exposure concentrations, exposure periods, imaging approaches, and uptake analyses across these models enables direct assessment of cell type‐dependent differences in MNP interactions. Accordingly, this study′s principal contribution is not identifying size‐dependent MNP uptake as a new phenomenon, but systematically comparing MNP behavior across multiple human cell types under standardized experimental conditions. The study further integrates time‐dependent uptake measurements, polydisperse particle mixtures, barrier‐function assessments, and pharmacological interrogation of selected endocytic pathways. This comparative, multitissue approach provides a framework for determining which MNP responses are shared across cell types and which depend strongly on cellular context.

## 2. Methods

### 2.1. Chemicals

Eagle′s minimal essential medium (EMEM), nonessential amino acids (NEAA), GlutaMAX, antibiotic‐antimycotic solution, epidermal growth factor (EGF), CellMask Green, Hoechst 33342, and PrestoBlue were obtained from Gibco (ThermoFisher Scientific, Waltham, Massachusetts, United States). MCDB131 medium, Williams′ E medium, hydrocortisone, hydrocortisone hemisuccinate, and paraformaldehyde were purchased from Sigma–Aldrich (St. Louis, Missouri, United States). Fetal bovine serum (FBS) and trypan blue were obtained from Corning (Corning, New York, United States). Dimethyl sulfoxide (DMSO) was purchased from Fisher BioReagents (Waltham, Massachusetts, United States). Fluorescent polystyrene particles were obtained from Spherotech (Lake Forest, Illinois, United States; catalog numbers FP‐0256‐2, FP‐0556‐2, FP‐0856‐2, and FP‐1552‐2). According to the manufacturer, the respective nominal size ranges were 0.25, 0.54, 0.87, and 1.12 *μ*m. We did not independently determine hydrodynamic diameter, zeta potential, or particle‐size distribution in the different culture media used in this study. We calculated approximate particle number concentrations from the nominal particle diameter and mass concentration, assuming spherical particles and a polystyrene density of 1.05 g/cm^3^. These values represent theoretical estimates and do not account for particle‐size distribution, aggregation, or changes in hydrodynamic diameter in culture medium. ProLong Diamond Antifade Mounting Medium with DAPI was purchased from Molecular Probes (ThermoFisher Scientific).

### 2.2. Cell Culture Conditions

Caco‐2 cells were maintained in EMEM supplemented with 1% NEAA and 20% FBS [49]. The same basal medium was used for MRC‐5 [50], HMC‐3 [51], and HEK293‐hTLR2 cells [52], with FBS reduced to 10%. The human epidermal keratinocytes HEK001 were maintained in a keratinocyte‐serum‐free medium supplemented with 5 ng/mL human recombinant EGF [53]. HMEC‐1 cells were cultured in MCDB131 medium supplemented with 10 ng/mL EGF, 1 *μ*g/mL hydrocortisone, 10 mM L‐glutamine, and 10% FBS [54]. Undifferentiated HepaRG cells were grown in Williams′ E medium containing 1% GlutaMAX, 5 *μ*g/mL insulin, 50 *μ*M hydrocortisone hemisuccinate, and 10% FBS, whereas differentiation was induced by supplementing the growth medium with 1.7% DMSO [55] (Table 1). All culture media were additionally supplemented with 1% antibiotic‐antimycotic, and cells were maintained at 37°C in a humidified atmosphere with 5% CO_2_. All experiments were conducted using cells within defined passage ranges to minimize variability associated with prolonged culture. Passage numbers were P7‐P10 for HEK001, P27‐P38 for Caco‐2, P21‐P23 for MRC‐5, P23‐P25 for HEK293‐hTLR2, P18‐P20 for HepaRG, P15‐P20 for HMC‐3, and P20‐P25 for HMEC‐1 cells (Table 1). Particle exposures were conducted in the complete culture medium specified for each cell model. Because medium composition and serum concentration differed among cell models, medium‐dependent changes in particle surface properties, protein‐corona formation, sedimentation, and aggregation cannot be excluded. Experiments were conducted using three independent biological replicates, with each treatment evaluated in four technical replicate wells within each experiment.

**Table 1:** Human cell lines used in this study.

Cell line	Tissue type	Cell type	Passage range	Provider	Culture media	Reference
HEK001	Skin	Keratinocytes	P7–P10	ATCC	Keratinocyte‐serum free Medium +5 ng/mL human recombinant EGF	[53]
Caco‐2	Colon	Enterocytes	P27–P38	ATCC	EMEM + 20% FBS	[49]
MRC‐5	Lung	Fibroblast	P21–P23	ATCC	EMEM + 10% FBS	[50]
HEK293‐hTLR2	Kidney	Epithelial	P23–P25	InvivoGen	EMEM + 10% FBS	[52]
HepaRG	Liver	Hepatocytes	P18–P20	BioPredic International	William′s E Medium + 2 mM GlutaMAX + 10% FBS +5 *μ*g/mL insulin + 50 *μ*M hydrocortisone hemisuccinate	[55]
HMC‐3	Brain	Microglial	P15–P20	ATCC	EMEM + 10% FBS	[51]
HMEC‐1	Vascular	Endothelial	P20–P25	ATCC	MCDB131 medium + 10 ng/ml EGF + 1 *μ*g/mL hydrocortisone +10 mM glutamine	[54]

Abbreviations: ATCC, American Type Culture Collection; EMEM, Eagle′s minimal essential medium; FBS, fetal bovine serum.

### 2.3. Cytotoxicity

Cells were seeded into 96‐well plates at a density of 75,000–100,000 cells/mL and allowed to attach and stabilize for 24 h, or until reaching approximately 50%–60% confluency to ensure cells were in the exponential growth phase at the time of exposure. Following stabilization, cells were exposed to MNPs of defined sizes (0.04, 0.25, 0.54, 0.87, and 1.12 *μ*m) or to a polydisperse mixture containing all five‐particle sizes at equal mass fractions (1:1:1:1:1). The reported exposure concentrations for the mixture refer to the total polymer mass concentration rather than the concentration of each individual particle‐size fraction. Accordingly, each fraction contributed 20% of the total administered mass (e.g., 20 *μ*g/mL of each particle size for a total mixture concentration of 100 *μ*g/mL). This mass‐based approach ensured equivalent total polymer concentrations between individual particle‐size treatments and the polydisperse mixture. Particles were administered at total concentrations ranging from 0.01 to 100 *μ*g/mL (0.01, 0.1, 1, 10, and 100 *μ*g/mL) to characterize concentration‐dependent cellular responses across a broad experimental range. A 20% DMSO treatment was included as a positive control to induce maximal cytotoxicity and validate assay performance. Cell‐free wells containing MNPs at the corresponding concentrations in culture medium were included as particle‐only controls to assess potential interference with the assay. No appreciable particle‐associated interference was detected under the assay conditions. Cells were exposed for 24 or 48 h to evaluate short‐term cellular responses. Cell viability was assessed using the PrestoBlue resazurin‐based metabolic assay, which measures the reducing capacity of viable cells as an indicator of mitochondrial activity [56, 57]. Because PrestoBlue measures metabolic reducing activity rather than cell number directly, increases in assay signal were not interpreted as direct evidence of cell proliferation. Four hours prior to the end of each exposure period, 10 *μ*L of PrestoBlue reagent was added directly to each well without removing the exposure media, minimizing disturbance to the cells. Plates were then returned to the incubator to allow sufficient time for resazurin to be reduced to the fluorescent resorufin product. Fluorescence (excitation: 560 nm; emission: 590 nm) and absorbance (570 nm) were subsequently measured using a Thermo Varioskan LUX multimode plate reader.

### 2.4. Live‐Cell Imaging and Temporal Analysis of NP Exposure

For live‐cell imaging experiments, cells were seeded into 96‐well plates at a density of 100,000–150,000 cells/mL and allowed to adhere for 24 h, or until reaching approximately 60%–70% confluency to ensure optimal cell morphology and coverage for imaging. Following this stabilization period, cells were exposed to MNPs at a concentration of 100 *μ*g/mL. This concentration was selected as an experimental condition to maximize particle visibility and tracking under the imaging conditions of the BioTek Lionheart FX Automated Microscope while maintaining cell viability during the observation period; it was not intended to represent an environmentally realistic exposure concentration. Because of the imaging system′s optical resolution limitations, the smallest particle size (0.04 *μ*m) could not be reliably detected and was therefore excluded from live imaging analyses. Immediately after MNP addition, plates were transferred to the microscope stage equipped with environmental control (37°C, 5% CO_2_) to maintain physiological conditions throughout the experiment. Time‐lapse images were acquired at 30 min intervals over a total duration of 48 h, allowing continuous monitoring of NP‐cell interactions, particle association, and potential internalization dynamics. Image acquisition and analysis were performed using Gen5 Image Prime Software. Sequential images were compiled into time‐lapse videos to visualize temporal changes in NP distribution and cellular responses. This approach enabled qualitative assessment of particle‐cell interactions over time, providing complementary insight into uptake kinetics and behavior that cannot be captured through endpoint assays alone.

### 2.5. Fluorescence Imaging of NP Uptake

Eight‐well chamber slides were seeded with cells at a density of 100,000–150,000 cells/mL and allowed to attach and stabilize for 24 h, or until reaching approximately 60%–70% confluency. Cells were then exposed to Nile Red‐labeled MNPs of 0.25, 0.54, 0.87, or 1.12 *μ*m at a concentration of 10 *μ*g/mL for 24 or 48 h. We selected this concentration to provide sufficient particle‐associated fluorescence for imaging while maintaining cell viability. Particle aggregation was not quantitatively characterized under these conditions. Furthermore, fluorescence stability, potential Nile Red leaching, and fluorescence intensity per particle or unit mass were not independently evaluated across particle sizes. Consequently, differences in RFU/cell among particle sizes may reflect variations in particle labeling and fluorescence properties in addition to differences in cell‐associated particle abundance. We excluded the smallest NP size (0.04 *μ*m) because confocal laser scanning microscopy has limited resolution. Following exposure, cells were washed 2–3 times with phosphate‐buffered saline (PBS) to remove free particles. Cell membranes were stained using CellMask Green, a fluorescent plasma membrane dye, and cells were subsequently fixed with 4% paraformaldehyde. The chamber was removed by immersing the slide in an alcohol bath for 10–15 min. After drying, a mounting medium containing 4 ^′^,6‐diamidino‐2‐phenylindole (DAPI), a fluorescent nuclear stain that binds DNA, was applied to label nuclei and secure the coverslip. Fluorescence images were acquired using an Olympus FV3000 Confocal Laser Scanning Microscope with DAPI (blue), fluorescein isothiocyanate (FITC; green), and tetramethylrhodamine (TRITC; red) filter sets. Relative fluorescence intensity was quantified using cellSens Imaging Software to assess particle‐associated signal with cells. Because the imaging and fluorescence quantification used in this study did not unequivocally distinguish membrane‐associated particles from intracellular particles, RFU/cell was interpreted as a measure of cell‐associated particle fluorescence rather than exclusively intracellular uptake.

### 2.6. Endocytosis Inhibition

To investigate MNP internalization mechanisms, we used pharmacological inhibitors targeting specific endocytic pathways under exposure conditions consistent with those described in the previous cytotoxicity, live‐cell imaging, and fluorescent imaging experiments, unless otherwise noted. Cytochalasin D was used to inhibit actin‐dependent processes by disrupting actin filament polymerization. Because cytochalasin D is not selective for a single actin‐dependent uptake mechanism, its effects were interpreted as evidence for the involvement of actin‐dependent processes rather than as specific evidence of phagocytosis or macropinocytosis. Cells were coexposed to MNPs and 1 *μ*M cytochalasin D for 48 h during live‐cell imaging, whereas quantitative fluorescence analysis was restricted to 24 h. During live‐cell imaging, morphological evidence of inhibitor‐associated cytotoxicity became apparent after approximately 30 h in most cell models. Therefore, observations beyond 30 h were considered qualitative and were not used to support mechanistic conclusions. The 24‐h fluorescence endpoint was selected to evaluate MNP‐cell association before overt morphological evidence of cytochalasin D‐associated cytotoxicity was observed. Cytochalasin D‐only cytotoxicity was not independently quantified using a cell viability assay under the 24‐h conditions employed for fluorescence analysis. Therefore, the selection of this endpoint was based on the absence of overt morphological evidence of cytotoxicity during live‐cell imaging. Nevertheless, potential sublethal or metabolic effects of cytochalasin D cannot be excluded.

Clathrin‐mediated endocytosis (CME) was inhibited using Pitstop‐2, a small‐molecule inhibitor that blocks the terminal domain of clathrin, preventing the assembly of clathrin‐coated pits and vesicles [58]. Cells were pre‐treated with 30 *μ*M Pitstop‐2 for 30 min prior to MNP exposure, followed by coexposure to MNPs for 2 h to specifically target early uptake events. MNP concentrations were adjusted depending on the imaging modality to optimize data quality: for live‐cell imaging, the concentration was reduced to 50 *μ*g/mL to minimize background signal and improve visualization, whereas for confocal laser scanning microscopy analysis, the concentration was increased to 100 *μ*g/mL to enhance fluorescence intensity and allow detection of more subtle differences in uptake.

Transferrin conjugated to Alexa Fluor 633 (Transferrin‐Alexa633), which is internalized predominantly through CME, was used as a positive control to verify the inhibitory activity of Pitstop‐2 against a well‐established CME‐associated process under the experimental conditions. This control was used to confirm inhibitor activity and was not interpreted as direct evidence that MNP association or internalization occurred exclusively through CME. Following treatment, cells were processed and imaged according to the procedures described in the live‐cell imaging and fluorescent imaging sections. This inhibitor‐based approach was used to evaluate whether the observed MNP‐cell interactions were consistent with contributions from specific endocytic processes across different cell types.

### 2.7. Membrane Damage Assay

Membrane integrity was assessed using a modified protocol based on Perry et al. [59]. HMEC‐1 and Caco‐2 cells were seeded onto 8‐well chamber slides at a density of 150,000 cells/mL and allowed to adhere and stabilize for 24 h, or until reaching approximately 60%–70% confluency. Cells were exposed to MNPs under conditions optimized for each cell type. HMEC‐1 cells were treated with 10 *μ*g/mL to minimize cytotoxicity and avoid reduced imaging resolution due to excessive particle burden. In contrast, Caco‐2 cells, which did not exhibit these limitations, were exposed to 100 *μ*g/mL MNPs to maximize detectable effects. All exposures were conducted for 48 h. A 10–15 min treatment with 100 *μ*M Triton X‐100, a membrane‐disrupting detergent, was included as a positive control for membrane damage. Following exposure, cells were washed twice with PBS and incubated with trypan blue for 5 min to label cells with compromised membrane integrity. Cells were then fixed with 4% paraformaldehyde for 20 min at room temperature and washed 2–3 times with PBS. Nuclear staining was performed using Hoechst 33342 (8 *μ*M) for 15 min to enable accurate cell counting, followed by two final PBS washes. Cells were immediately imaged using the BioTek Lionheart FX Automated Microscope under phase‐contrast, color brightfield, and DAPI channels. Image analysis was conducted using Gen5 Image Prime Software, where blue‐channel intensity (trypan blue uptake) and total cell counts were quantified to assess membrane damage relative to controls.

### 2.8. Barrier Integrity Evaluation

HMEC‐1 and Caco‐2 cells were seeded onto 24‐well Transwell inserts at a density of 150,000 cells/mL and maintained for 4–7 days to allow the formation of a confluent monolayer. Barrier integrity was monitored by measuring transepithelial/transendothelial electrical resistance (TEER), an indicator of tight junction formation and permeability, using an EVOM2 Epithelial Voltohmmeter equipped with STX electrodes. TEER measurements were recorded every 24 h until a stable plateau was reached, indicating the establishment of a functional barrier. Once stable TEER values were achieved, cells were exposed to noncytotoxic concentrations of MNPs and incubated at 37°C with 5% CO_2_ for 48 h. A 100 *μ*M Triton X‐100 treatment was included as a positive control to induce barrier disruption. Following exposure, cells were gently washed 2–3 times with PBS to remove residual particles, and final TEER measurements were obtained. Raw resistance values (*Ω*) were corrected by subtracting blank (cell‐free insert) measurements and normalized to the membrane surface area (*Ω*·cm^2^). Changes in barrier integrity were assessed by comparing pre‐exposure (baseline) and postexposure TEER values for each well. This approach enabled the evaluation of MNP‐induced alterations in epithelial and endothelial barrier function.

### 2.9. Statistical Analysis

All statistical analyses were performed using GraphPad Prism 10 (GraphPad Software, San Diego, California). Experiments consisted of three independent biological replicates, with four technical replicates per treatment within each biological replicate. Technical replicates were averaged within each biological replicate and were not treated as independent observations; therefore, the biological replicate was considered the independent statistical unit (*n* = 3). Data were evaluated for normality and homogeneity of variance using the Shapiro–Wilk and Brown–Forsythe tests, respectively. When assumptions of normality and equal variance were met, data were analyzed using one‐way analysis of variance (ANOVA) followed by either Dunnett′s multiple comparisons test for comparisons against the negative control or Sidak′s multiple comparisons test for pairwise comparisons among groups. Student′s *t*‐test was used for direct comparisons between two groups, including comparisons between 24 and 48 h or between the presence and absence of cytochalasin D, as indicated in the corresponding figure legends. When assumptions of normality and equal variance were not met, we analyzed data using the Kruskal–Wallis test followed by Dunn′s multiple comparisons test. Statistical significance was defined as *p* < 0.05, unless otherwise specified. Additionally, cytotoxicity concentration‐response curves were fitted by nonlinear regression in GraphPad Prism 10 to estimate EC_50_ values. When the fitted EC_50_ exceeded the highest experimentally tested concentration (100 *μ*g/mL), the value was considered an extrapolated model estimate rather than an experimentally resolved EC_50_.

## 3. Results

### 3.1. Cytotoxicity

Across all tested cell lines (HEK001, Caco‐2, MRC‐5, HEK293‐hTLR2, HepaRG, HMC‐3, and HMEC‐1), no cytotoxicity was observed after 24 h of exposure to MNPs at any concentration tested (0.01–100 *μ*g/mL), with cell viability remaining comparable to controls. After 48 h, viability was generally unchanged or significantly increased at higher doses (100 *μ*g/mL). These increases are interpreted as enhanced metabolic reducing activity rather than evidence of cytotoxicity. For instance, HEK001 cells reached 145 ± 29.2% with 1.12 *μ*m NPs, whereas Caco‐2 cells increased to 125 ± 5.9% and 111 ± 17.3% with 0.87 *μ*m and 0.25 *μ*m NPs, respectively. Similar trends were observed in HEK293‐hTLR2 and HMEC‐1 cells, as well as with the MNP mixture. Viability decreased modestly only in HMEC‐1 cells at 48 h after exposure to the largest particles and the MNP mixture. The estimated EC_50_ values were 121.8 ± 18.3 and 92.16 ± 20.05 * μ*g/mL, respectively; however, because the calculated values extended beyond or approached the upper end of the tested concentration range (100 *μ*g/mL), they should be interpreted cautiously as model‐derived estimates rather than directly measured EC_50_ values. Overall, these results indicate no toxicity at 24 h and suggest increased metabolic reducing activity at 48 h under higher MNP exposures. We calculated theoretical particle number concentrations assuming spherical particles and a polystyrene density of 1.05 g/cm^3^. At 100 *μ*g/mL, the estimated particle numbers were 2.84 × 10^12^, 1.16 × 10^10^, 1.16 × 10^9^, 2.76 × 10^8^, and 1.29 × 10^8^ particles/mL for the 0.04, 0.25, 0.54, 0.87, and 1.12 *μ*m particles, respectively. Particle numbers decreased proportionally with mass concentration across the tested range of 0.01–100 *μ*g/mL. Thus, equivalent mass concentrations produced substantial differences in particle number across particle sizes, with the estimated number of 0.04 *μ*m particles approximately 22,000‐fold greater than that of 1.12 *μ*m particles.

### 3.2. Live‐Cell Analysis of NP Exposure

Across all models, negative control wells showed normal cell growth with minimal background interference, whereas all MNP‐exposed groups exhibited high initial background signal at 0 h due to particle scattering and reflection. Tissue‐specific differences in MNP interaction and accumulation were evident over time.

Epithelial cell models showed distinct responses. Skin keratinocytes (HEK001) displayed rapid and pronounced MNP interaction, with edge‐associated accumulation by 6 h and strong aggregation for 0.25–0.54 *μ*m particles. By 12 h, larger particles (1.12 *μ*m) were nearly cleared from the background and internalized, and by 24–48 h, extracellular MNPs were qualitatively reduced, with decreasing aggregate size despite persistence in daughter cells (Figure 1). In contrast, intestinal epithelial cells (Caco‐2) showed minimal interaction with smaller particles (0.25–0.87 *μ*m) over 48 h, with only delayed, limited accumulation of 1.12 *μ*m and mixed MNPs at 24–48 h and no clear aggregate formation (Figure S1). Fibroblast cells (MRC‐5, lung) demonstrated moderate and size‐dependent MNP interactions. Accumulation at cell edges was evident by 6 h for 0.54–1.12 *μ*m particles, followed by intracellular aggregation and partial clearance of background MNPs between 12–24 h. By 48 h, MNP distribution reflected cell migration paths. Smaller particles (0.25 *μ*m) showed delayed accumulation without pronounced intracellular darkening, and mixed MNPs were less efficiently processed (Figure 2). Kidney‐derived cells (HEK293‐hTLR2) exhibited sustained accumulation and aggregation of MNPs. Edge‐associated accumulation occurred by 6 h for 0.54–1.12 *μ*m particles, with large aggregates forming by 12 h and persisting through 48 h. MNP‐depleted tracks were evident during live‐cell imaging and coincided with cell movement and changes in particle distribution. In contrast, smaller particles and mixtures exhibited weaker, less organized aggregation (Figure S2). Hepatic cells (HepaRG) exhibited delayed and relatively low MNP interaction. Minimal accumulation was observed up to 12 h, with only modest uptake of 0.54–1.12 *μ*m and mixed MNPs at 24–48 h. Interaction with smaller particles (0.25 *μ*m) remained negligible, with lower cell‐associated NP accumulation than that observed in several of the other cell models (Figure S3). Neural cells (HMC‐3, microglia) displayed strong size‐dependent responses. Early edge darkening was observed at 6 h for 0.87–1.12 *μ*m particles, followed by pronounced aggregation and substantial reduction of background MNPs by 48 h. Smaller particles and mixtures showed less distinct edge accumulation but were progressively internalized and transported with cells (Figure 3). Endothelial cells (HMEC‐1, vascular) exhibited dynamic but heterogeneous MNP interactions. Early accumulation was most evident for 1.12 *μ*m particles at 6 h, with high deposition but limited internalization between 6–12 h. By 24–48 h, all MNP sizes formed cell‐associated aggregates, although optical properties varied with particle size. Smaller particles (0.25–0.54 *μ*m) showed delayed accumulation without early edge darkening (Figure S4).

**Figure 1:**
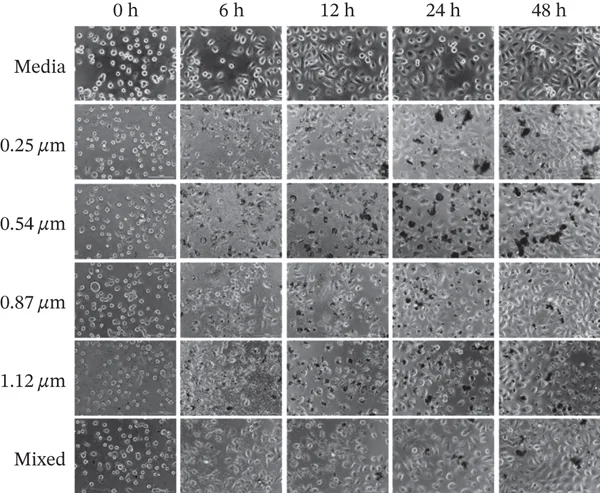
Live‐cell imaging of HEK001 cells exposed to 100 *μ*g/mL nanoplastics (NPs) of the indicated sizes for 48 h. NP sizes are listed on the left, and imaging time points (hours) are shown at the top. During the 0–6 h period, NPs appear as diffuse background noise in the treatment groups. Between 6 and 12 h, NPs begin to settle at the bottom of the wells and are generally visible as small black dots, although light reflection may cause some particles to appear white. As accumulation increases over time, NPs form larger dark aggregates or masses. Scale bar = 100 * μ*m.

**Figure 2:**
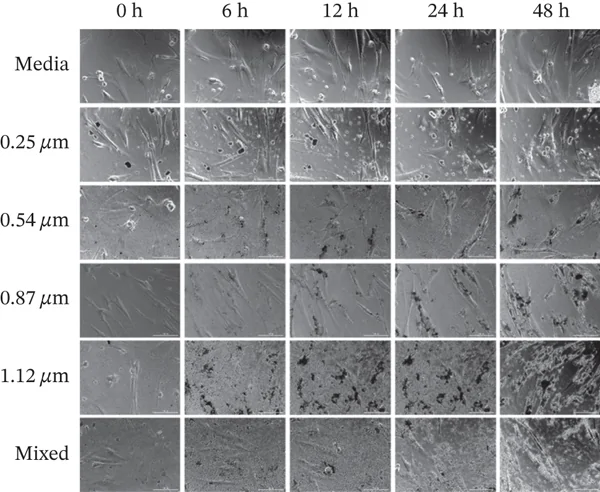
Live‐cell imaging of MRC‐5 cells exposed to 100 *μ*g/mL nanoplastics (NPs) of the indicated sizes for 48 h. NP sizes are listed on the left, and imaging time points (hours) are shown at the top. During the 0–6 h period, NPs appear as diffuse background noise in the treatment groups. Between 6 and 12 h, NPs begin to settle at the bottom of the wells and are generally visible as small black dots, although light reflection may cause some particles to appear white. As accumulation increases over time, NPs form larger dark aggregates or masses. Scale bar = 100 *μ*m.

**Figure 3:**
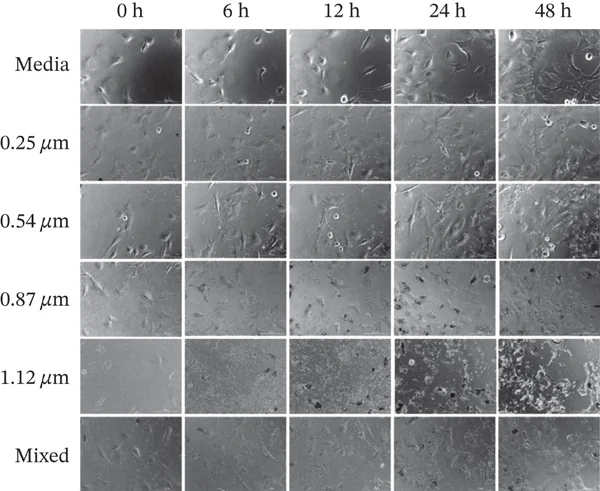
Live‐cell imaging of HMC‐3 cells exposed to 100 *μ*g/mL nanoplastics (NPs) of the indicated sizes for 48 h. NP sizes are listed on the left, and imaging time points (hours) are shown at the top. During the 0–6 h period, NPs appear as diffuse background noise in the treatment groups. Between 6 and 12 h, NPs begin to settle at the bottom of the wells and are generally visible as small black dots, although light reflection may cause some particles to appear white. As accumulation increases over time, NPs form larger dark aggregates or masses. Scale bar = 100 * μ*m.

Overall, MNP‐cell interactions were strongly dependent on cell type and particle size. HEK001 and HMC‐3 cells exhibited relatively rapid and extensive cell‐associated MNP accumulation, MRC‐5 and HEK293‐hTLR2 cells showed moderate and sustained accumulation, HMEC‐1 cells displayed delayed but dynamic interactions, and Caco‐2 and HepaRG cells showed comparatively lower cell‐associated MNP signals under the tested conditions. Across several cell types, directional cell movement toward MNPs and membrane extensions associated with particle‐cell interactions were observed.

### 3.3. Fluorescence Imaging of Cell‐Associated NPs

Qualitatively, there is a strong signal from the MNPs among most of the cells over 48 h with indications of size preferences by cell type (Figure 4). Semiquantitative cell‐associated MNP fluorescence, expressed as RFU/cell, showed clear cell‐type‐ and particle‐size‐dependent differences following 24 and 48 h exposure (Figure 5). In HEK001 keratinocytes, cell‐associated MNP fluorescence was significantly increased after 24 h for all tested particle sizes (*p* < 0.01). However, no significant differences were observed between 24 and 48 h, suggesting that most measurable cell‐associated fluorescence occurred within the first 24 h and remained relatively stable thereafter (Figure 5A). In contrast, Caco‐2 intestinal epithelial cells showed no significant MNP‐associated fluorescence compared with controls, and RFU/cell values remained relatively stable across particle sizes and exposure times (Figure 5B). MRC‐5 lung fibroblasts showed significant time‐dependent accumulation, with RFU/cell values increasing significantly from 24 to 48 h for all tested particle sizes (*p* < 0.01), indicating a continued increase in cell‐associated MNP fluorescence over the exposure period (Figure 5C). HEK293‐hTLR2 kidney cells also showed significant increases in MNP‐associated fluorescence at 48 h compared with 24 h for most particle sizes (*p* < 0.01), with the exception of 0.87 *μ*m NPs (Figure 5D). HepaRG hepatocytes displayed a strongly size‐dependent response. Significant increased cell‐associated fluorescence was observed for the larger 0.87 and 1.12 *μ*m MNPs (*p* < 0.01), whereas 0.54 *μ*m NPs showed lower accumulation at 48 h. No significant increase in cell‐associated fluorescence was detected for 0.25 *μ*m NPs (Figure 5E). HMC‐3 microglial cells also exhibited size‐dependent and time‐dependent responses, with greater increases from 24 to 48 h for the larger 0.87 and 1.12 *μ*m MNPs and the MNP mixture, whereas smaller temporal changes were observed for 0.25 and 0.54 *μ*m NPs (Figure 5F). In HMEC‐1 endothelial cells, all tested MNP sizes produced significantly elevated RFU/cell values at 24 h, whereas at 48 h significant accumulation was maintained for 1.12 *μ*m MNPs. However, no significant differences were detected between the 24‐ and 48‐h time points for any particle size (Figure 5G). Overall, cell‐associated MNP fluorescence depended strongly on both the cellular model and particle size. Some cell types, such as HEK001 and HMEC‐1, showed significant accumulation by 24 h with little additional change at 48 h, whereas MRC‐5 and HEK293‐hTLR2 cells generally showed continued accumulation over time. Larger MNPs (0.87–1.12 *μ*m) tended to produce higher cell‐associated fluorescence signals in several cell models, particularly HepaRG and HMC‐3, whereas Caco‐2 cells exhibited comparatively low fluorescence signals under the tested conditions. However, these differences should not be interpreted as direct quantitative differences in particle uptake because fluorescence intensity was not independently standardized across particle sizes. Although the mixed particles produced a signal that was greater than controls, only HMC‐3 and HEK293‐hTLR2 increased accumulation over time (Figure S5).

**Figure 4:**
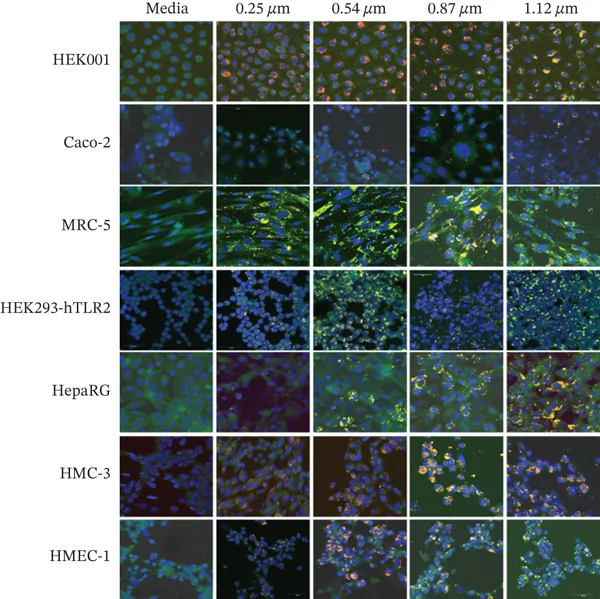
Fluorescence imaging of all cell models after 48 h exposure to 10 *μ*g/mL nanoplastics (NPs) of the indicated sizes. Nanoplastics are shown in yellow or red, cell nuclei in blue, and cell membranes in green. Tissue and size‐dependent differences in NP accumulation and aggregation were observed across cell types, with prominent intracellular clustering in several models. Scale bar = 100 * μ*m.

**Figure 5:**
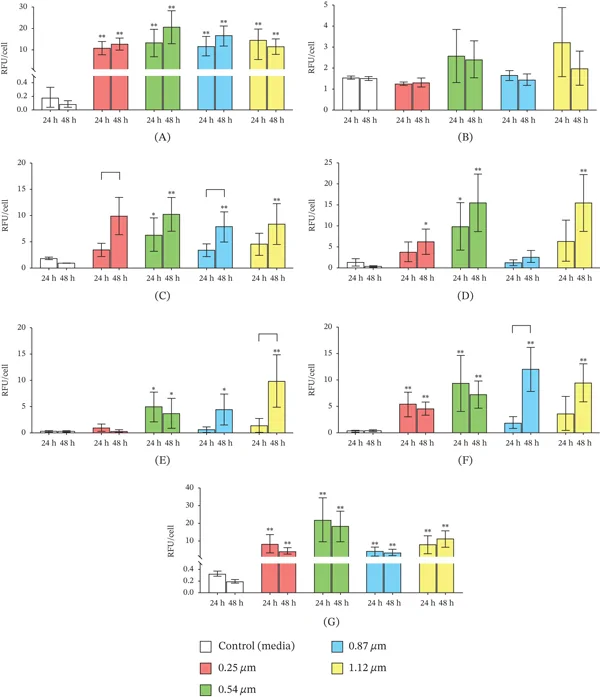
Nanoplastic (NP) cell‐associated fluorescence, expressed as relative fluorescent units (RFU)/cell, following 24 h and 48 h exposure to 10 *μ*g/mL NPs of the indicated sizes (0.25, 0.54, 0.87, and 1.12 *μ*m) in all cell models: (A) HEK001 keratinocytes, (B) Caco‐2 intestinal epithelial cells, (C) MRC‐5 lung fibroblasts, (D) HEK293‐hTLR2 kidney cells, (E) HepaRG hepatocytes, (F) HMC‐3 microglia, and (G) HMEC‐1 endothelial cells. Data are presented as mean ± SD from three independent biological replicates (*n* = 3), each containing four technical replicates. Technical replicates were averaged within each biological replicate before statistical analysis. Statistical significance against the corresponding control groups is indicated as  ^∗^ *p* < 0.05 and  ^∗∗^ *p* < 0.01 (one‐way ANOVA, Dunnett′s multiple comparisons test). Black brackets denote statistically significant differences between 24 h and 48 h within the same experimental condition, as determined by Student′s *t*‐test (*p* < 0.05).

### 3.4. Endocytosis Inhibition

Cytochalasin D treatment inhibited cell motility and division during the first 24 h across the evaluated cell models. Overt morphological evidence of cytotoxicity, including membrane blebbing and rupture, became apparent after approximately 30 h in most cell lines, except HMC‐3 microglia. Consequently, quantitative fluorescence analysis was restricted to the 24‐h endpoint, whereas observations beyond 30 h were considered potentially confounded by inhibitor‐associated cytotoxicity and were excluded from mechanistic interpretation. During the noncytotoxic observation period, HEK001 keratinocytes showed visible MNP aggregation with cells, with reduced cell‐associated MNP accumulation in the presence of cytochalasin D, whereas Caco‐2 intestinal epithelial cells exhibited minimal MNP interaction overall. MRC‐5 lung fibroblasts and HMC‐3 microglia also exhibited reduced MNP association, although residual particle clustering around cells suggested that some particle‐cell interactions may occur independently of actin polymerization. In contrast, HEK293‐hTLR2 kidney cells and HMEC‐1 endothelial cells showed strong inhibition of MNP interaction, with very limited cell‐associated MNP accumulation. HepaRG hepatocytes exhibited almost complete suppression of MNP interaction under cytochalasin D exposure conditions (Figure S6).

Semi‐quantitative RFU/cell analysis at 24 h, prior to the appearance of overt morphological cytotoxicity, supported these observations (Figure 6). Cytochalasin D exposure significantly reduced cell‐associated fluorescence from 1.12 *μ*m MNPs in HEK001 (Figure 6A), HEK293‐hTLR2 (Figure 6D), HMC‐3 (Figure 6F), and HMEC‐1 (Figure 6G) cells (*p* < 0.01), with notable decreases in MRC‐5 fibroblasts (Figure 6C). For 0.87 *μ*m NPs, RFU/cell significantly decreased in HEK001 (Figure 6A), HEK293‐hTLR2 (Figure 6D), HMC‐3 (Figure 6F), cells (*p* < 0.01), whereas MRC‐5 fibroblasts showed a modest increase. Cell‐associated fluorescence from 0.54 *μ*m NPs was significantly reduced in HEK001 (*p* < 0.05), HMC‐3 (*p* < 0.05), HepaRG (*p* < 0.01), HEK293‐hTLR2 (*p* < 0.01), and HMEC‐1 cells (*p* < 0.01). No statistically significant decreases were observed for 0.25 *μ*m or mixed MNP (Figure S5) exposures across most cell lines. Interestingly, Caco‐2 intestinal epithelial cells (Figure 6B) showed a trend toward increased RFU/cell values during cytochalasin D exposure, suggesting that MNP association in this model may rely less strongly on actin‐dependent uptake pathways.

**Figure 6:**
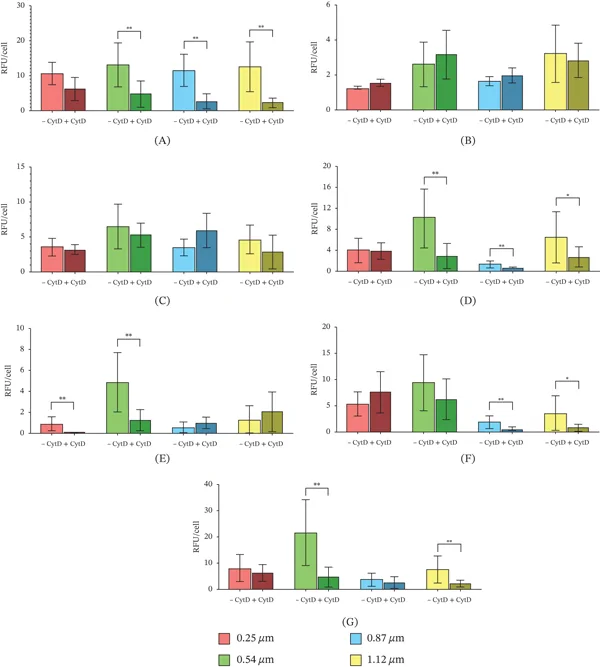
Nanoplastic (NP) cell‐associated fluorescence, expressed as relative fluorescent units (RFU)/cell, following exposure to 10 *μ*g/mL NPs of the indicated sizes (0.25, 0.54, 0.87, and 1.12 *μ*m) in the presence or absence of cytochalasin D (+ CytD and − CytD, respectively) in all cell models: (A) HEK001, (B) Caco‐2, (C) MRC‐5, (D) HEK293‐hTLR2, (E) HepaRG, (F) HMC‐3, and (G) HMEC‐1 cells. Data are presented as mean ± SD from three independent biological replicates (*n* = 3), each containing four technical replicates. Technical replicates were averaged within each biological replicate before statistical analysis. Black brackets denote statistically significant differences between the presence and absence of cytochalasin D within the same experimental condition, as determined by Student′s *t*‐test ( ^∗^ *p* < 0.05 and  ^∗∗^ *p* < 0.01).

Exposure to Pitstop‐2 revealed cell and particle size‐dependent changes in MNP‐associated fluorescence that were consistent with a contribution of CME in selected cell models. HEK001 cells showed a marked reduction in MNP interaction across all sizes during live imaging, with particles remaining largely at the bottom of the well over 2 h, and a significant decrease in RFU/cell across all treatments (*p* < 0.01) (Figure S7). In contrast, MRC‐5 (Figure S8), HEK293‐hTLR2 (Figure S9), and HMEC‐1 (Figure S10) cells showed limited observable differences in live imaging, although fluorescent quantification revealed selective effects. MRC‐5 cells showed significant decreases in RFU/cell for 0.25 *μ*m and 0.54 *μ*m NPs, whereas HEK293‐hTLR2 cells showed a decrease for 0.25 *μ*m (*p* <0.01) but an increase for 1.12 *μ*m MNPs (*p* <0.01). HMEC‐1 cells showed a significant decrease only for 0.54 *μ*m NPs (*p* < 0.01). Caco‐2 and HepaRG cells were not included in the Pitstop‐2 analysis because their relatively slow and limited MNP interactions during the short 2‐h inhibitor exposure period resulted in insufficient particle‐associated signal for reliable assessment of inhibition. HMC‐3 cells were excluded because Pitstop‐2 treatment produced an unexpected increase in RFU/cell rather than the anticipated decrease, preventing reliable interpretation of CME inhibition and suggesting a potential cell‐specific off‐target or nonspecific effect of the inhibitor. Overall, the inhibition studies support size and cell type‐dependent differences in MNP‐cell interactions, with the cytochalasin D results indicating a greater contribution of actin‐dependent processes to the association of larger particles, whereas the Pitstop‐2 experiments support a cell‐ and size‐dependent contribution of CME.

### 3.5. Barrier Integrity Evaluation

Consistent with the limited cytotoxicity observed under inhibitor conditions, barrier integrity and membrane permeability remained unaffected in epithelial and endothelial models. Trypan blue exclusion assays showed no significant membrane damage in either HMEC‐1 (vascular endothelium) or Caco‐2 (intestinal epithelium) following MNP exposure (Figure S11). Similarly, TEER measurements revealed no significant differences between pre‐exposure and postexposure values after 48 h, indicating preserved barrier function in both cell types.

## 4. Discussion

The present study extends previous work on size‐dependent MNP uptake by systematically comparing MNP interactions across seven human cell models using a common experimental design. Rather than establishing size‐dependent uptake as a novel phenomenon, our findings show how uptake dynamics and cellular responses vary among tissues when particle characteristics and exposure conditions are held constant. Across all models, the absence of cytotoxicity at 24 h and the general increase in viability/metabolism at 48 h indicate that, under the tested conditions (0.01–100 *μ*g/mL), MNPs did not induce acute toxicity but were associated with increased metabolic reducing activity in some cell models. This is consistent with previous studies reporting minimal cytotoxicity across a range of NP sizes and polymers in relevant cell lines [15, 16, 27, 37, 60–64]. However, the increased PrestoBlue signal observed in HEK001, Caco‐2, and HEK293‐hTLR2 cells at 48 h, particularly at higher concentrations, suggests a potential proliferative or stress‐adaptive response rather than a purely inert interaction. In keratinocytes (HEK001), this response may be linked to cytokine‐mediated signaling, as IL‐1 and IL‐6 are known to promote keratinocyte proliferation [65], and similar pro‐inflammatory signaling has been reported in lung and gut models following NP exposure [27, 66]. Conversely, cytotoxic effects reported in other studies, particularly with larger particles, higher doses, or surface‐functionalized MNPs [26, 67], highlight the importance of physicochemical properties such as surface charge and concentration. The modest decrease in viability observed in HMEC‐1 cells with 1.12 *μ*m and mixed MNPs further supports emerging evidence that endothelial cells may exhibit selective sensitivity to larger or polydisperse particles [68–70], suggesting that toxicity is not solely governed by particle size but also by tissue‐specific susceptibility.

A central finding of this study is the strong dependence of MNP interaction on tissue origin. Keratinocytes (HEK001) and microglia (HMC‐3) exhibited rapid and extensive MNP association, whereas intestinal (Caco‐2) and hepatic (HepaRG) cells showed comparatively limited interaction. The rapid reduction of extracellular MNPs observed in HEK001 cultures and their preferential association with 0.54–0.87 *μ*m particles are consistent with previous keratinocyte studies that have demonstrated enhanced uptake of mid‐sized particles [30]. This may reflect size‐dependent differences in particle number, surface area, and cell‐particle interactions, as smaller particles are more numerous at equivalent mass concentrations [71], potentially leading to rapid saturation or carrying capacity limits at 10 *μ*g/mL. Indeed, the lack of increased fluorescence per cell for 0.25 *μ*m NPs in HEK001 and HMC‐3 cells could be consistent with an apparent plateau in cell‐associated particle signal; however, saturation was not directly evaluated, and alternative explanations cannot be excluded. In contrast, the minimal interaction observed in Caco‐2 cells contrasts with numerous reports of MNP uptake in this model [15, 60, 63, 64]. This discrepancy likely reflects methodological differences. Flow cytometry‐based quantification, commonly used in the literature, cannot distinguish between surface‐bound and internalized particles without additional processing [72], and qualitative imaging from prior studies often reveals heterogeneous uptake, with only subsets of cells exhibiting visible MNP accumulation. Passage number may also contribute to variability; although the present study used passages 27–38, previous work indicates that even moderate increases in passage can alter permeability and transport properties [73, 74]. These findings suggest that stricter standardization of passage number and validation with primary or iPSC‐derived intestinal models may be necessary to reduce variability and improve reproducibility in MNP research.

Fibroblasts (MRC‐5) and kidney‐derived cells (HEK293‐hTLR2) demonstrated robust and sustained MNP association across multiple sizes, supporting their roles as active responders to environmental stressors. The broad uptake profile observed in HEK293‐hTLR2 cells aligns with previous studies reporting interactions with diverse polymers and particle sizes [24, 75], suggesting a relatively nonselective, “generalist” endocytic capacity. In fibroblasts, the strong affinity for micron‐scale particles is consistent with Raman microscopy observations in similar cell types [76] and may have important implications for pulmonary exposure. Although smaller particles (≤ 100 nm) are traditionally considered more hazardous due to deep lung penetration [19], disease states such as COPD, asthma, and cancer can compromise epithelial and endothelial barriers, increasing permeability and facilitating the retention of larger particles [77–79]. Given the role of fibroblasts in regulating inflammation and extracellular matrix remodeling, their strong interactions with MNPs, known to induce pro‐inflammatory responses [7], raise important questions about their contribution to chronic lung pathology under combined exposure and disease conditions.

Hepatic (HepaRG) and endothelial (HMEC‐1) cells exhibited more selective and dynamic MNP interactions. HepaRG cells showed delayed MNP association, with significant accumulation of 0.87 *μ*m and 1.12 *μ*m particles and reduced interaction with smaller or mixed MNPs, consistent with previous reports indicating size and surface‐dependent uptake in hepatocytes [25, 62]. The observed decrease in RFU/cell for 0.54 *μ*m particles over time may reflect several processes, including particle release, redistribution, or dilution through cell division. Although exocytosis has been described as a potential mechanism of particle clearance [80], this process was not directly evaluated in the present study. Therefore, the observed reduction in fluorescence cannot be attributed to a specific mechanism. Similarly, HMEC‐1 cells displayed highly dynamic and size‐specific responses, with increased association of larger particles (1.12 *μ*m), decreased accumulation of smaller particles (0.25–0.54 *μ*m), and no change for intermediate sizes. These patterns may reflect a combination of rapid uptake followed by exocytosis and dilution through cell division [81]. The selective sensitivity of endothelial cells to specific particle sizes is supported by previous studies in HUVEC models [45, 69, 82] and may be linked to the high density of caveolae and specialized transport functions in vascular endothelium [43].

Endocytosis inhibitors demonstrated that MNP uptake is governed by multiple, overlapping pathways that vary by particle size and cell type. Consistent with established size ranges [47, 83–85], cytochalasin D significantly reduced cell‐associated MNP fluorescence for several particle sizes and cell models at the 24‐h endpoint, supporting the involvement of actin‐dependent processes in MNP‐cell interactions, particularly for larger particles (0.54–1.12 *μ*m). However, because cytochalasin D broadly disrupts actin polymerization and is not selective for individual actin‐dependent uptake pathways, these findings cannot distinguish among specific mechanisms such as phagocytosis or macropinocytosis. Furthermore, although quantitative fluorescence analysis was restricted to 24 h to minimize potential interference from overt inhibitor‐associated cytotoxicity, the absence of morphological alterations does not exclude sublethal or metabolic effects. Because inhibitor‐only cytotoxicity was not independently quantified at this endpoint, the observed changes in RFU/cell should be interpreted cautiously as supportive, rather than definitive, evidence of actin‐dependent MNP‐cell interactions. For example, the lack of cytochalasin D inhibition for 0.87 *μ*m NPs in HepaRG and HMEC‐1 cells may reflect low baseline accumulation at 24 h, limiting detection of inhibitory effects. The increased RFU/cell observed in Caco‐2 cells under cytochalasin D treatment may indicate altered membrane permeability due to cytoskeletal disruption, as actin filaments are critical for maintaining structure. Notably, most of the cell lines failed to exhibit actin‐dependent uptake for 0.25 *μ*m or mixed MNPs, suggesting reliance on alternative pathways such as CME or caveolin‐mediated uptake. Pitstop‐2 experiments produced cell‐ and particle size‐dependent reductions in MNP‐associated fluorescence that were consistent with a contribution of CME. In particular, the responses observed in MRC‐5 and HEK293‐hTLR2 cells were consistent with a contribution of CME to interactions with smaller particles, whereas the HMEC‐1 response suggested a possible CME contribution for intermediate‐sized particles (0.54 *μ*m). However, because these conclusions are based on a single pharmacological inhibitor, they should not be interpreted as definitive evidence of CME. Confirmation using complementary pharmacological or genetic approaches would be required to establish the specific involvement of this pathway. The high density of caveolae in endothelial cells [43] suggests that nonclathrin‐dependent pathways may also contribute to MNP‐cell interactions in this model. The strong inhibition observed in HEK001 cells across all MNP sizes was unexpected and may reflect broader clathrin involvement or off‐target effects of Pitstop‐2, highlighting the need for validation using additional inhibitors such as dynasore, chlorpromazine, or phenylarsine oxide [48, 86, 87].

An additional factor that may contribute to the apparent size‐dependent uptake is particle sedimentation. Live‐cell imaging showed particles progressively settling toward the bottom of the wells during exposure. Because all cell models were adherent, sedimentation may increase particle contact with the cell monolayer and therefore influence the effective cellular dose. Differences in sedimentation among particle sizes could consequently contribute to the observed differences in cellular association and uptake, independently of, or in addition to, intrinsic size‐dependent endocytic mechanisms. Because sedimentation rates and delivered cellular doses were not quantitatively determined, the observed size‐dependent responses should be interpreted in the context of the combined effects of particle size, particle number, and particle behavior under the exposure conditions.

This study also compared monodisperse and polydisperse MNP exposures across multiple human cell models. Across most cell types, mixed MNP treatments produced lower cell‐associated fluorescence signals than individual particle‐size treatments, with significant time‐dependent increases observed only in HEK293‐hTLR2 and HMC‐3 cells. The mechanisms underlying these differences remain unclear. Variations in particle behavior, aggregation, competition among particle sizes, or the involvement of multiple cellular processes could potentially contribute to the observed responses; however, these possibilities remain speculative because they were not directly investigated. Furthermore, differences in fluorescence properties among particle sizes may influence comparisons between monodisperse and polydisperse treatments. Future studies incorporating particle characterization, fluorescence calibration, and complementary uptake measurements will be necessary to clarify the mechanisms underlying these observations.

Despite these advances, several limitations should be acknowledged. First, the use of immortalized cell lines may not fully capture the complexity of in vivo systems, including multicellular interactions, extracellular matrix effects, and physiological flow conditions. Second, variability in passage number, differentiation state, and culture conditions may influence uptake and should be more tightly controlled and reported across studies. Third, although RFU/cell provides a normalized, semiquantitative measure of cell‐associated MNP fluorescence, the imaging approach used here does not unequivocally distinguish membrane‐associated from intracellular particles. Furthermore, fluorescence intensity per particle or unit mass was not independently validated across particle sizes, and potential differences in Nile Red labeling, fluorescence stability, and dye leaching cannot be excluded. Therefore, differences in RFU/cell among particle sizes should not be interpreted as direct quantitative differences in particle uptake. Instead, these measurements should be considered indicators of cell‐associated fluorescence under the tested conditions. Although confocal imaging provides spatial information consistent with particle‐cell interactions, additional approaches would be required to confirm intracellular localization and reliably quantify particle uptake across different sizes. Finally, the study focused on short‐term exposures (≤ 48 h); longer‐term studies are needed to evaluate chronic effects, exocytosis dynamics, inflammatory responses, and functional consequences such as barrier disruption or immune activation. Additionally, hydrodynamic diameter, zeta potential, aggregation behavior, fluorescence stability, and particle‐size distribution were not independently characterized under the different culture conditions. Because medium composition and serum concentration varied among cell models, medium‐dependent changes in particle behavior cannot be excluded. Live‐cell imaging indicated particle settling and cell‐associated aggregation for some particle sizes and cell models; however, these observations were qualitative and should not be considered measurements of colloidal stability. Future studies should characterize these physicochemical properties directly under the relevant exposure conditions. An additional limitation is that particle sizes were compared with equivalent mass concentrations. Because particle number scales inversely with particle volume, equivalent mass concentrations resulted in substantially greater particle numbers and total particle surface area for the smaller particles. Thus, the observed size‐dependent responses may reflect differences in particle number and available surface area in addition to intrinsic effects of particle size. Future studies comparing exposures normalized by particle number or surface area would help distinguish these factors.

Overall, this study demonstrates that MNP uptake is a highly dynamic and cell‐type‐specific process governed by particle size, cellular physiology, and the availability of endocytic pathways. By evaluating seven human cell models using standardized particle sizes, exposure conditions, and analytical approaches, this study provides a comparative framework for distinguishing broadly shared MNP responses from those that are strongly dependent on cellular context. This multitissue comparison represents the principal added value of the study and highlights the limitations of extrapolating MNP uptake and cellular responses from a single cell model to other human tissues.

## 5. Conclusions

Overall, MNPs did not produce detectable cytotoxicity in most cell models under the tested conditions, although this finding should not be interpreted as an absence of other biological effects. The only exception was HMEC‐1 endothelial cells, which showed decreased viability following exposure to 1.12 *μ*m and mixed MNPs, suggesting that endothelial systems may be more sensitive to specific particle sizes and distributions. Cell‐associated MNP fluorescence varied across the 24‐ and 48‐h exposure periods, with distinct patterns among cell types and particle sizes. An apparent plateau in fluorescence was observed in HEK001 and HMC‐3 cells exposed to 0.25 *μ*m particles, although saturation was not directly investigated. Similarly, reductions in cell‐associated fluorescence observed in HMEC‐1 cells could reflect several processes, including particle release or dilution through cell division, but these mechanisms were not independently evaluated. Importantly, because fluorescence intensity was not standardized across particle sizes, these findings should be interpreted as differences in cell‐associated fluorescence rather than absolute quantitative differences in particle uptake. Pharmacological inhibition experiments were consistent with contributions from actin‐dependent processes and CME to MNP‐cell interactions, with the apparent contribution of these processes varying among cell types and particle sizes. In contrast, interactions with polydisperse MNP mixtures appeared more complex, potentially reflecting differences in particle behavior, aggregation, competition among particle sizes, or the involvement of multiple cellular processes. Collectively, these findings indicate that MNP‐cell interactions and cell‐associated fluorescence patterns vary among cell types and particle sizes. However, differences in fluorescence intensity should not be interpreted as absolute quantitative differences in particle uptake without additional validation. These findings highlight the importance of comparative multitissue approaches and complementary analytical methods for understanding MNP interactions with human cells and their potential implications for human health.

## Author Contributions

**Alisha M. Janiga-MacNelly:** conceptualization, formal analysis, investigation, methodology, validation, visualization, writing – original draft. **Ramon Lavado:** conceptualization, data curation, formal analysis, funding acquisition, project administration, supervision, validation, visualization, writing – review and editing.

## Funding

This study was supported by the Baylor University, 10.13039/100007492, C. Gus Glasscock, Jr. Endowed Fund of Excellence 2025.

## Disclosure

All scientific content, interpretations, and final edits were reviewed and approved by the authors.

## Conflicts of Interest

The authors declare no conflicts of interest.

## Supporting information


**Supporting Information** Additional supporting information can be found online in the Supporting Information section. Supplementary Information Figures S1–S11 provides additional data on micro‐ and nanoplastics (MNPs) interactions, cellular uptake, and endocytic mechanisms in human cell models. Live‐cell imaging documents MNP sedimentation, aggregation, and cellular interactions over 48 h (Figures S1–S4). Fluorescence analyses evaluate MNP accumulation and the effects of cytochalasin D (Figures S5, S6) and Pitstop‐2, including transferrin uptake controls to validate inhibition of clathrin‐mediated endocytosis (Figures S7–S10). Plasma membrane integrity following NP exposure was assessed using Trypan Blue staining (Figure S11).

## Data Availability

The data supporting the findings of this study are available from the corresponding author upon reasonable request.
