# Supplementary material for: Uptake of Micro‐ and Nano‐Sized Plastic Particles in Human Cells: Size‐Dependent Endocytosis and Cellular Responses
Source: Biomed Res Int. 2026 Oct 21;2026:4933959. doi: 10.1155/bmri/4933959 (PMC13687932; doi:10.1155/bmri/4933959)
Supplement: Supplementary file 1 [file BMRI-2026-4933959-s001.pdf]

Micro- and nano-sized plastic particles uptake in human cells: size-dependent endocytosis and cellular responses

Alisha M. Janiga-MacNelly and Ramon Lavado

School of Earth and Environmental Sciences, Baylor University, Waco, TX 76798

Supporting Information

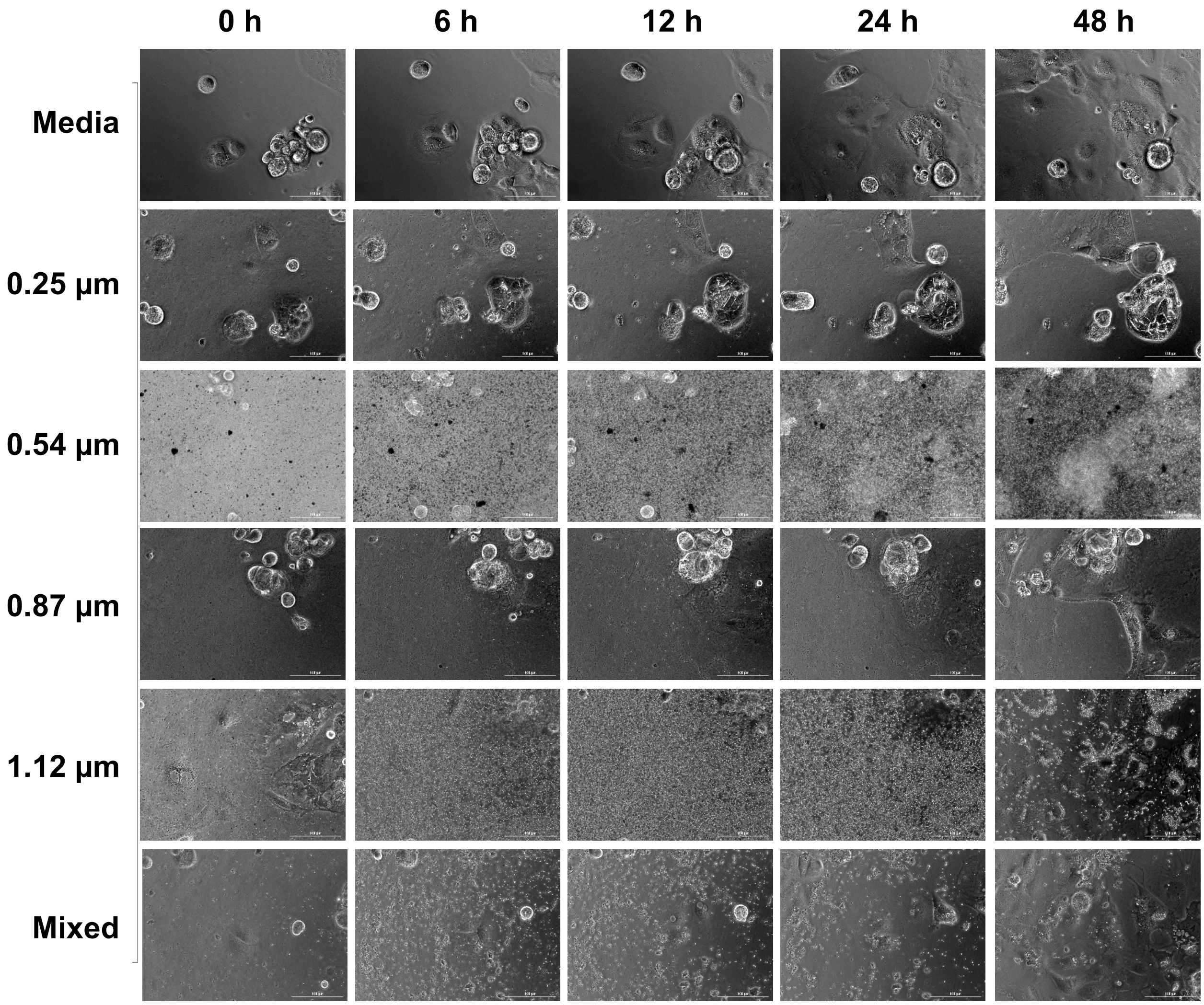

Figure S1. Live-cell imaging of Caco-2 cells exposed to 100 μg/mL nanoplastics (NPs) of the indicated sizes for 48 h. NP sizes are listed on the left, and imaging time points (hours) are shown at the top. During the 0-6 h period, NPs appear as diffuse background noise in the treatment groups. Between 6 and 12 h, NPs begin to settle at the bottom of the wells and are generally visible as small black dots, although light reflection may cause some particles to appear white. As accumulation increases over time, NPs form larger dark aggregates or masses. Scale bar = 100 μm.

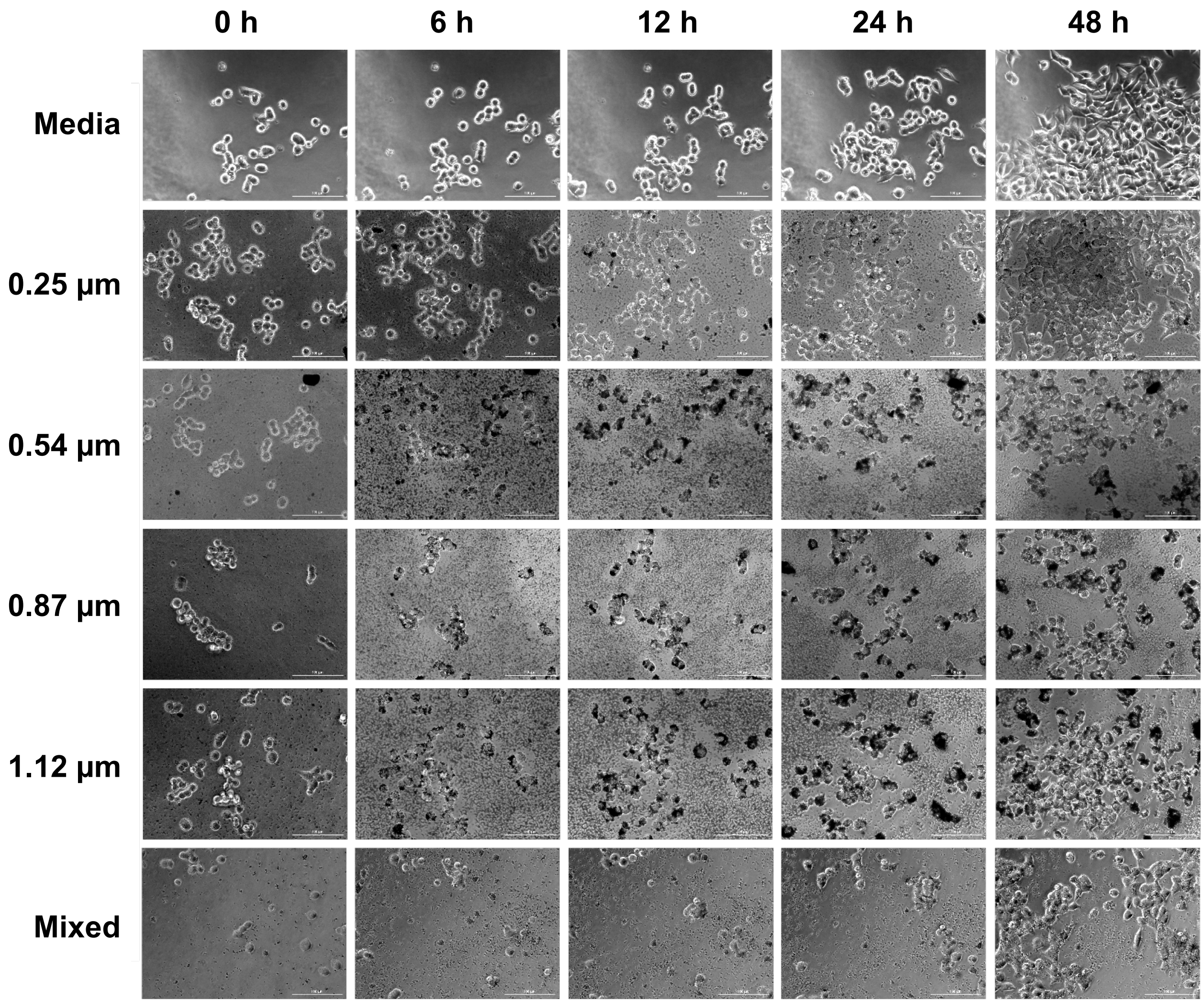

Figure S2. Live-cell imaging of HEK293-hTLR2 cells exposed to 100 μg/mL nanoplastics (NPs) of the indicated sizes for 48 h. NP sizes are listed on the left, and imaging time points (hours) are shown at the top. During the 0-6 h period, NPs appear as diffuse background noise in the treatment groups. Between 6 and 12 h, NPs begin to settle at the bottom of the wells and are generally visible as small black dots, although light reflection may cause some particles to appear white. As accumulation increases over time, NPs form larger dark aggregates or masses. Scale bar = 100 μm.

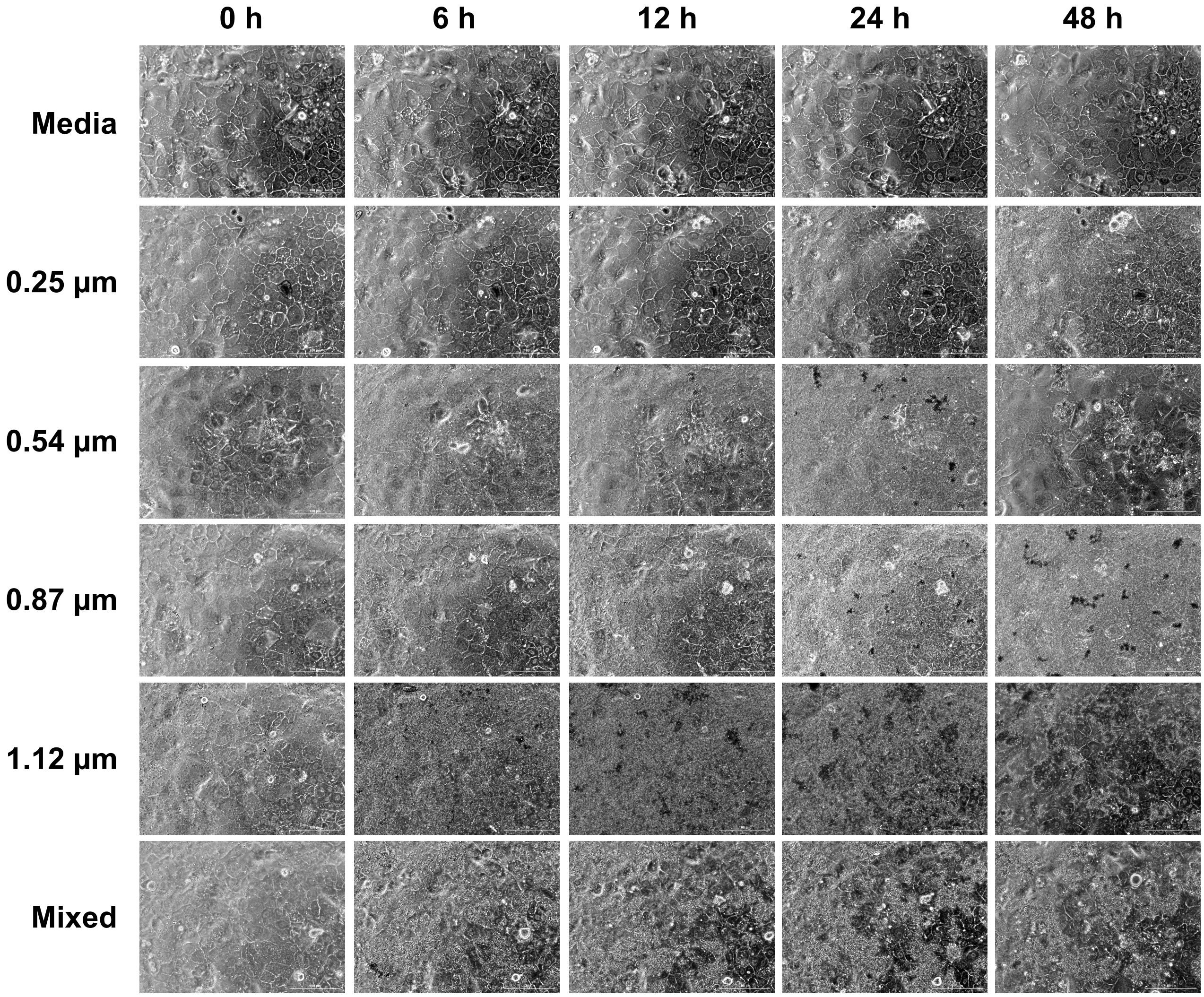

Figure S3. Live-cell imaging of HepaRG cells exposed to 100 μg/mL nanoplastics (NPs) of the indicated sizes for 48 h. NP sizes are listed on the left, and imaging time points (hours) are shown at the top. During the 0-6 h period, NPs appear as diffuse background noise in the treatment groups. Between 6 and 12 h, NPs begin to settle at the bottom of the wells and are generally visible as small black dots, although light reflection may cause some particles to appear white. As accumulation increases over time, NPs form larger dark aggregates or masses. Scale bar = 100 μm.

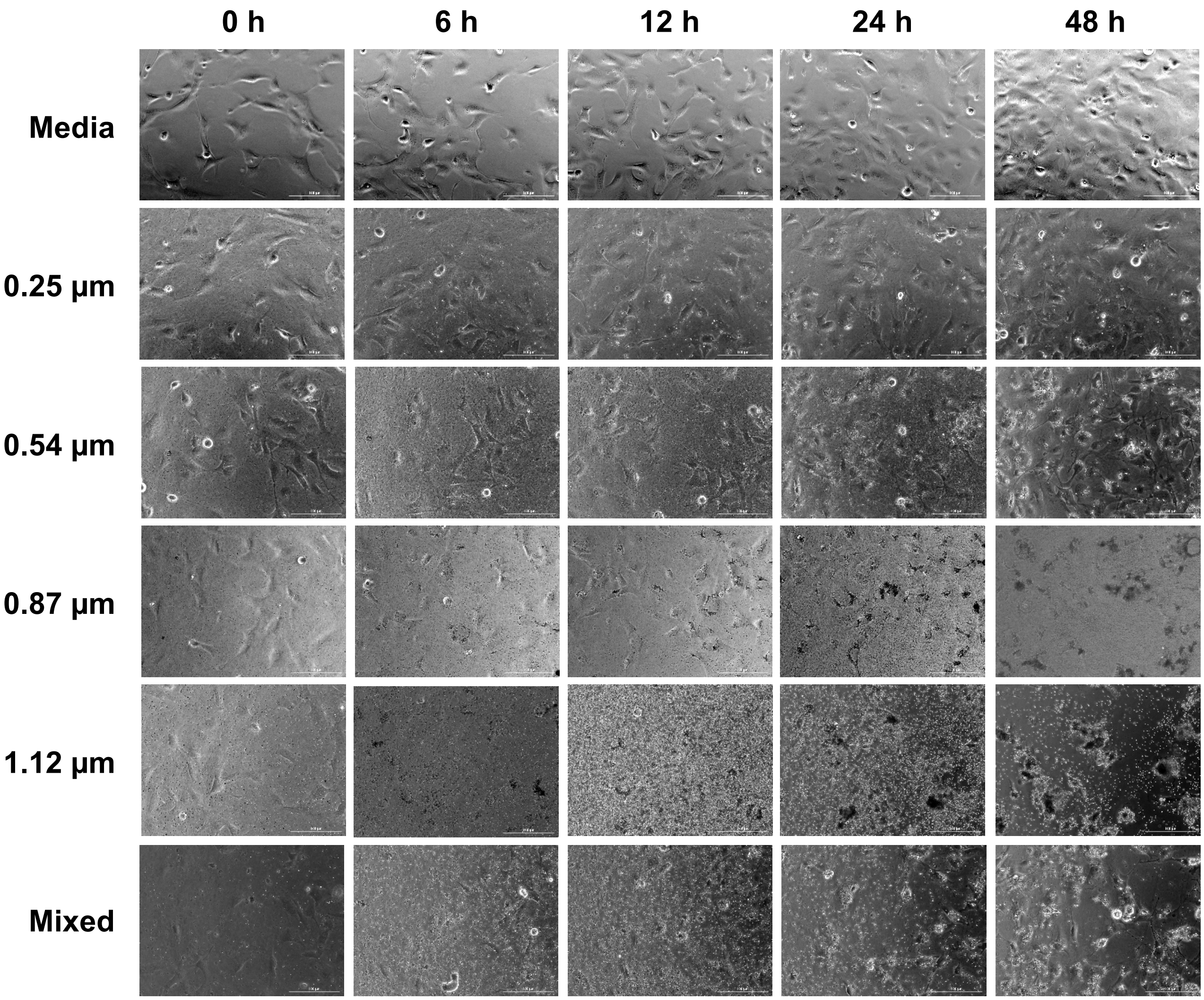

Figure S4. Live-cell imaging of HMEC-1 cells exposed to 100 µg/mL nanoplastics (NPs) of the indicated sizes for 48 h. NP sizes are listed on the left, and imaging time points (hours) are shown at the top. During the 0-6 h period, NPs appear as diffuse background noise in the treatment groups. Between 6 and 12 h, NPs begin to settle at the bottom of the wells and are generally visible as small black dots, although light reflection may cause some particles to appear white. As accumulation increases over time, NPs form larger dark aggregates or masses. Scale bar = 100 µm.

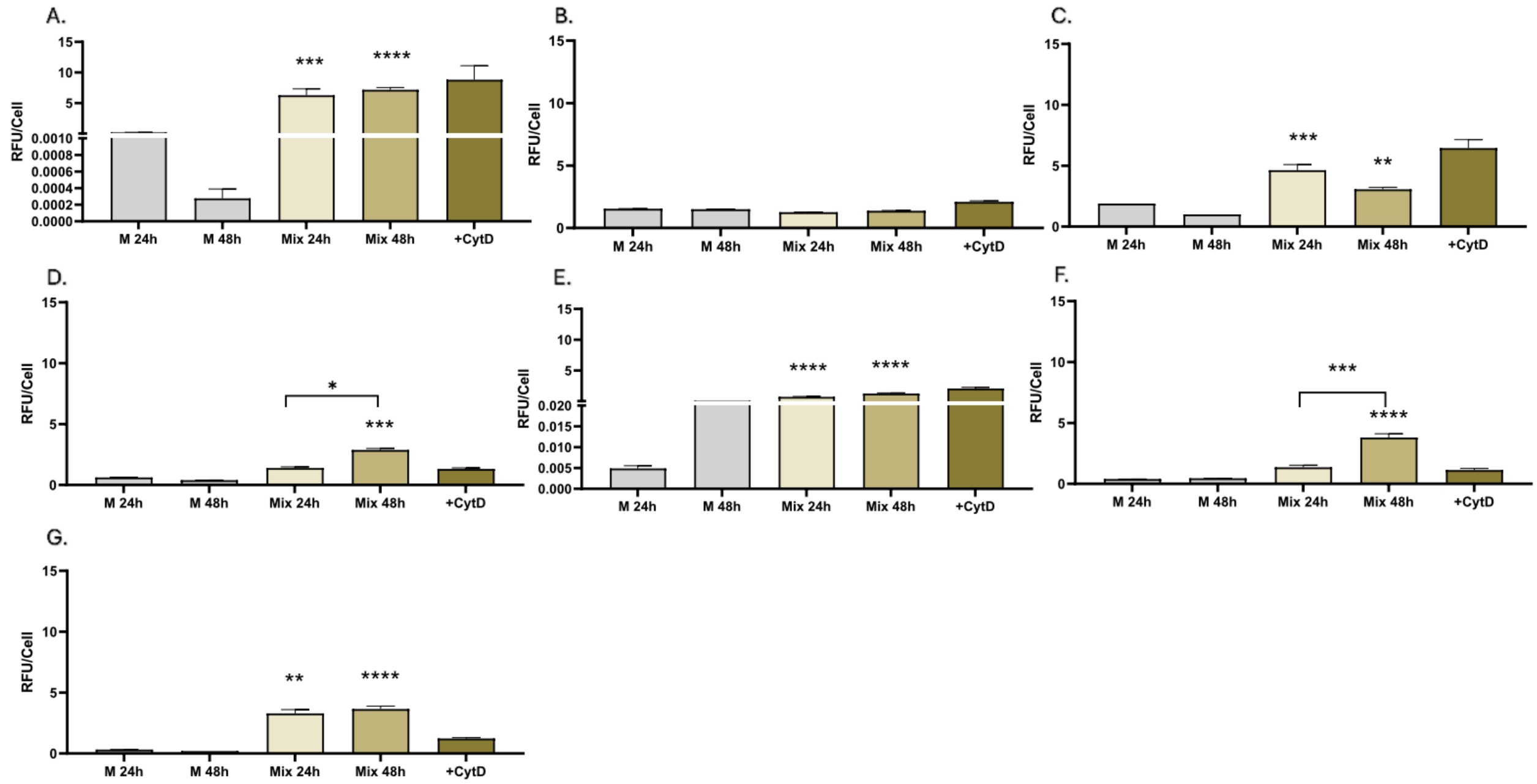

Figure S5. Nanoplastic (NP) cell-associated fluorescence, expressed as relative fluorescent units (RFU)/cell, following exposure to 10 µg/mL NPs of mixed sizes at 24 h, 48 h or in the presence of cytochalasin D (+CytD) in all cell models: HEK001 keratinocytes (A), Caco-2 intestinal epithelial cells (B), MRC-5 lung fibroblasts (C), HEK293-hTLR2 kidney cells (D), HepaRG hepatocytes (E), HMC-3 microglia (F), and HMEC-1 endothelial cells (G). Data are presented as mean ± SD from three independent biological replicates (n = 3), each containing four technical replicates. Technical replicates were averaged within each biological replicate before statistical analysis. Statistical significance against the corresponding control groups is indicated as * (one-way ANOVA, Dunnett's multiple comparisons test). Black brackets denote statistically significant differences between 24 h and 48 h within the same experimental condition (*$p < 0.05$, **$p < 0.01$, ***$p < 0.001$, ****$p < 0.0001$).

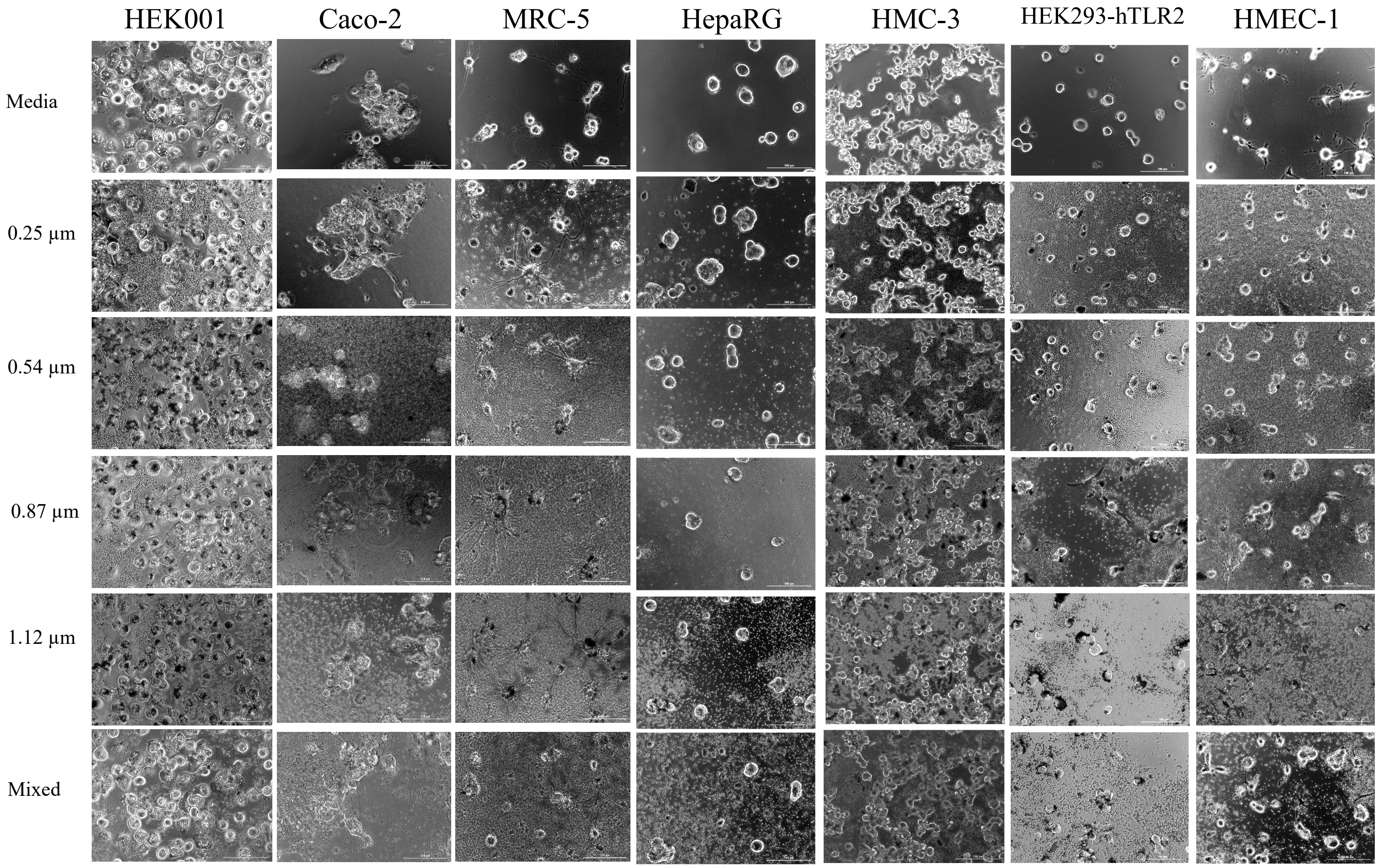

Figure S6. Live Cell Imaging of Cells with NPs and Cytochalasin D Cells were exposed to 100 μg/mL of NPs of sizes listed on the left side for 48 h along with 1 μM cytochalasin D. Scale bar = 100 μm

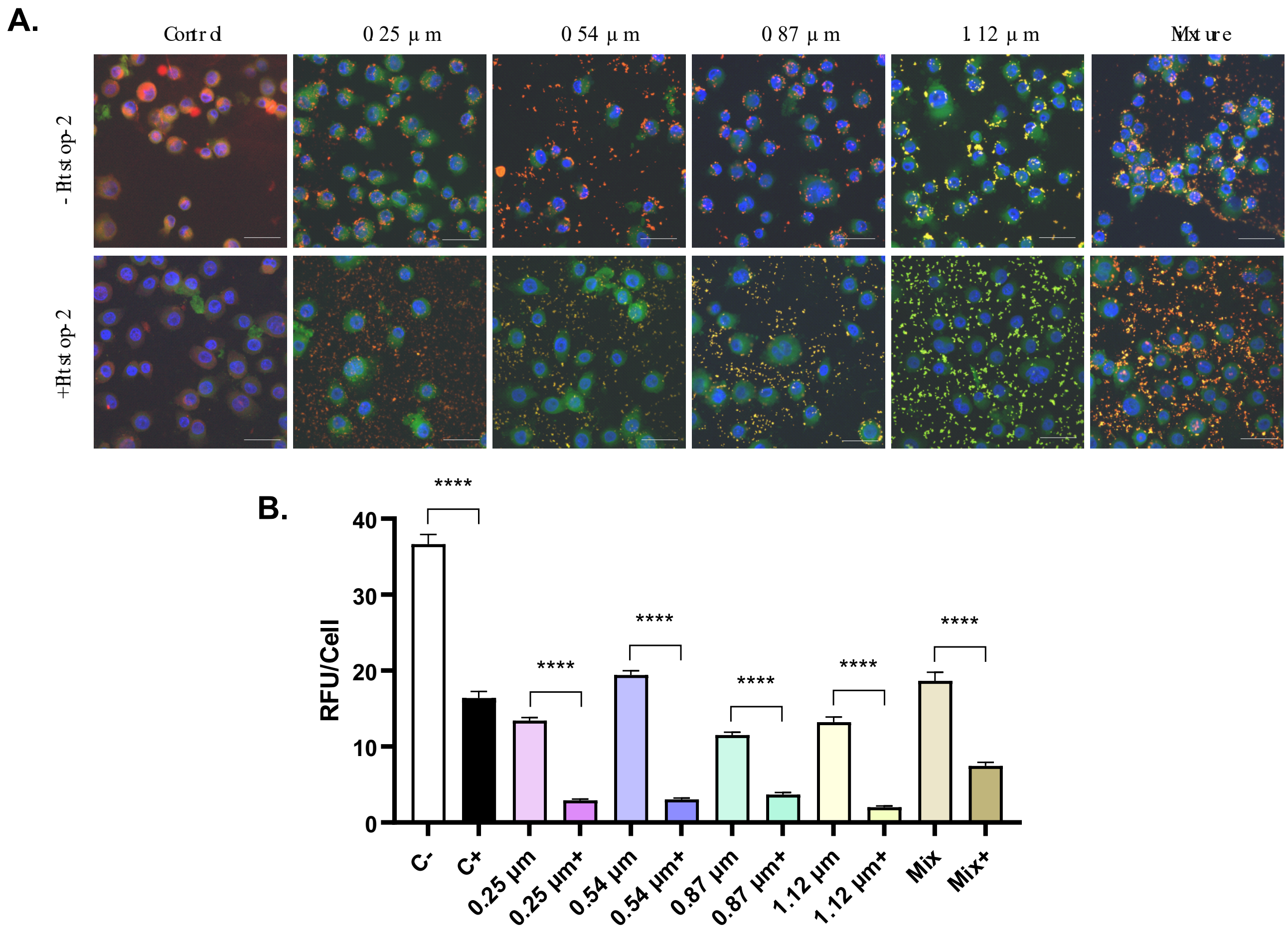

Figure S7. Fluorescence imaging of HEK001 cells after 2 h exposure to nanoplastics (NPs) at 10 µg/mL. (A) Representative fluorescence images of cells exposed to NPs alone or co-exposed with Pitstop-2 (+Pitstop-2). Transferrin-Alexa Fluor 633 was used as a positive control for clathrin-mediated endocytosis and was evaluated in the absence (C-) and presence (C+) of Pitstop-2 to confirm inhibition of transferrin uptake. NPs were stained with Nile Red, nuclei with DAPI, and cell membranes with CellMask Green. (B) Relative fluorescence units per cell (RFU/cell) following exposure to NPs alone or with Pitstop-2. Pitstop-2 significantly reduced transferrin-associated fluorescence (C+ vs. C-), confirming inhibition of transferrin uptake under the experimental conditions. Data were analyzed using one-way ANOVA followed by Sidak's multiple comparisons test (* p<0.05, ** p<0.01, *** p<0.001, **** p<0.0001). Error bars represent SEM. Scale bar = 40 µm.

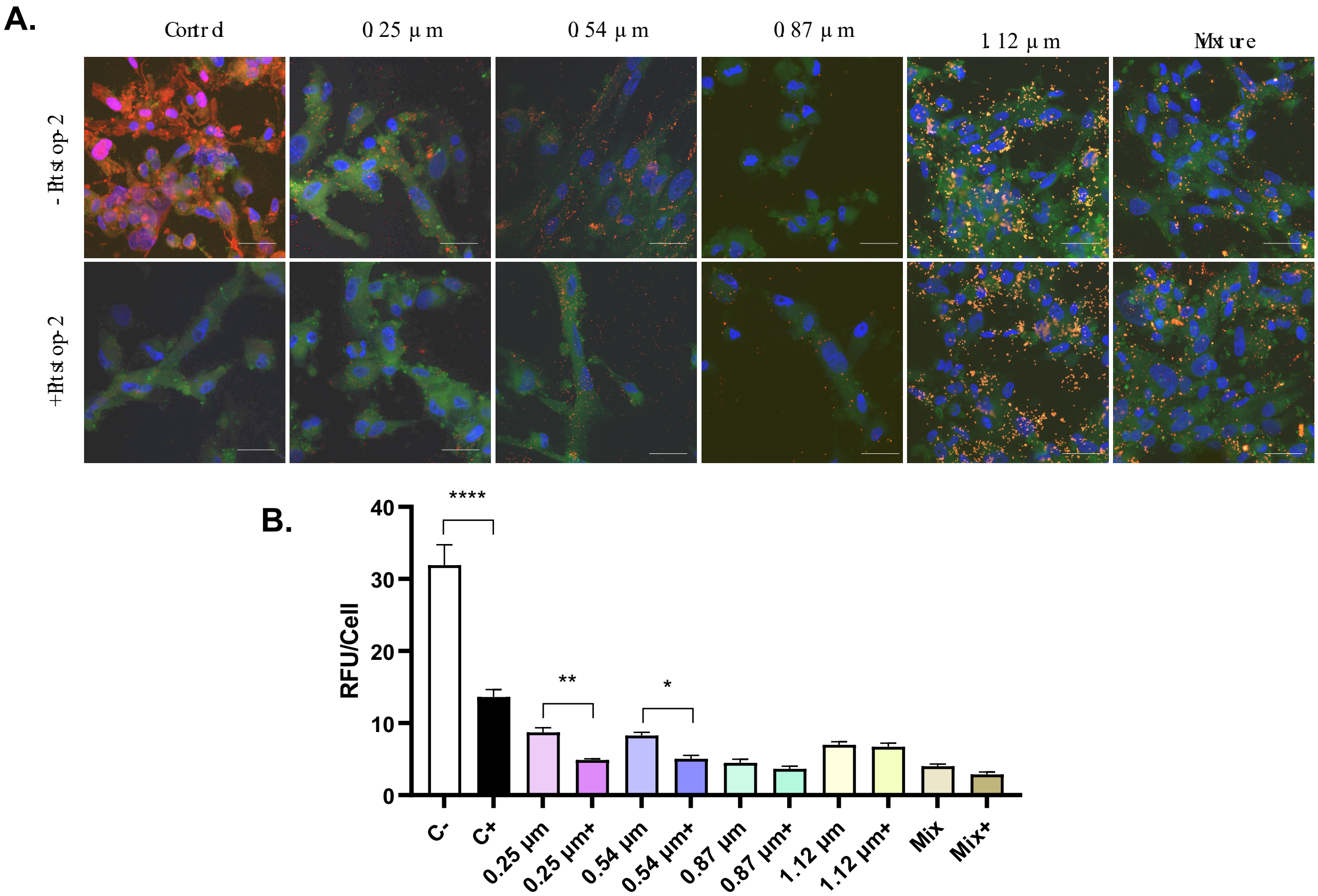

Figure S8. Fluorescence imaging of MRC-5 cells after 2 h exposure to nanoplastics (NPs) at 10 µg/mL. (**A**) Representative fluorescence images of cells exposed to NPs alone or co-exposed with Pitstop-2 (+Pitstop-2). Transferrin-Alexa Fluor 633 was used as a positive control for clathrin-mediated endocytosis and was evaluated in the absence (C-) and presence (C+) of Pitstop-2 to confirm inhibition of transferrin uptake. NPs were stained with Nile Red, nuclei with DAPI, and cell membranes with CellMask Green. (B) Relative fluorescence units per cell (RFU/cell) following exposure to NPs alone or with Pitstop-2. Pitstop-2 significantly reduced transferrin-associated fluorescence (C+ vs. C-), confirming inhibition of transferrin uptake under the experimental conditions. Data were analyzed using one-way ANOVA followed by Sidak's multiple comparisons test (* $p<0.05$, ** $p<0.01$, *** $p<0.001$, **** $p<0.0001$). Error bars represent SEM. Scale bar = 40 µm.

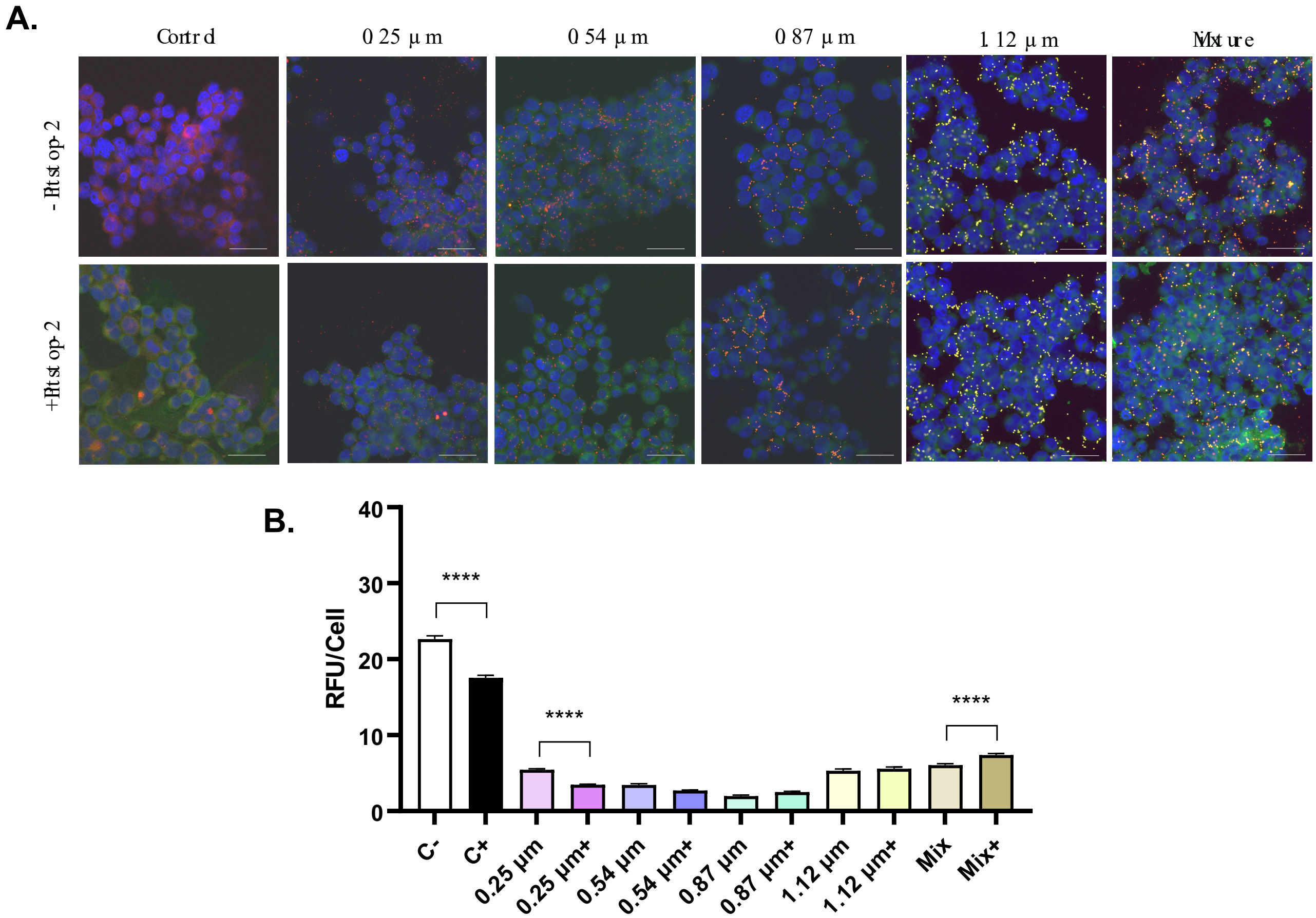

Figure S9. Fluorescence imaging of HEK293-hTLR2 cells after 2 h exposure to nanoplastics (NPs) at 10 µg/mL. (**A**) Representative fluorescence images of cells exposed to NPs alone or co-exposed with Pitstop-2 (+Pitstop-2). Transferrin-Alexa Fluor 633 was used as a positive control for clathrin-mediated endocytosis and was evaluated in the absence (C-) and presence (C+) of Pitstop-2 to confirm inhibition of transferrin uptake. NPs were stained with Nile Red, nuclei with DAPI, and cell membranes with CellMask Green. (B) Relative fluorescence units per cell (RFU/cell) following exposure to NPs alone or with Pitstop-2. Pitstop-2 significantly reduced transferrin-associated fluorescence (C+ vs. C-), confirming inhibition of transferrin uptake under the experimental conditions. Data were analyzed using one-way ANOVA followed by Sidak's multiple comparisons test (* p<0.05, ** p<0.01, *** p<0.001, **** p<0.0001). Error bars represent SEM. Scale bar = 40 µm.

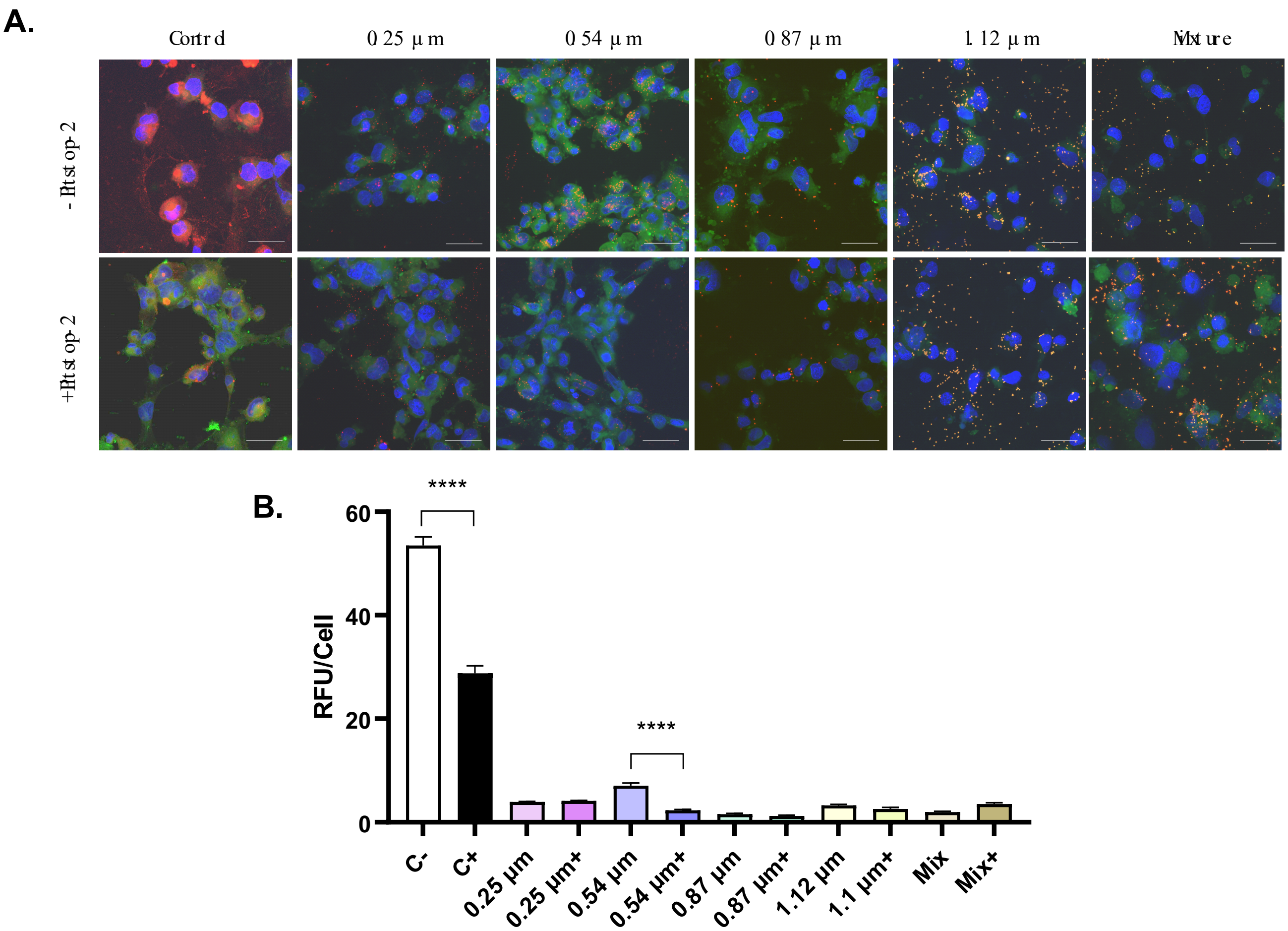

Figure S10. Fluorescence imaging of HMEC-1 cells after 2 h exposure to nanoplastics (NPs) at 10 µg/mL. (**A**) Representative fluorescence images of cells exposed to NPs alone or co-exposed with Pitstop-2 (+Pitstop-2). Transferrin-Alexa Fluor 633 was used as a positive control for clathrin-mediated endocytosis and was evaluated in the absence (C-) and presence (C+) of Pitstop-2 to confirm inhibition of transferrin uptake. NPs were stained with Nile Red, nuclei with DAPI, and cell membranes with CellMask Green. (B) Relative fluorescence units per cell (RFU/cell) following exposure to NPs alone or with Pitstop-2. Pitstop-2 significantly reduced transferrin-associated fluorescence (C+ vs. C-), confirming inhibition of transferrin uptake under the experimental conditions. Data were analyzed using one-way ANOVA followed by Sidak's multiple comparisons test (* p<0.05, ** p<0.01, *** p<0.001, **** p<0.0001). Error bars represent SEM. Scale bar = 40 µm.

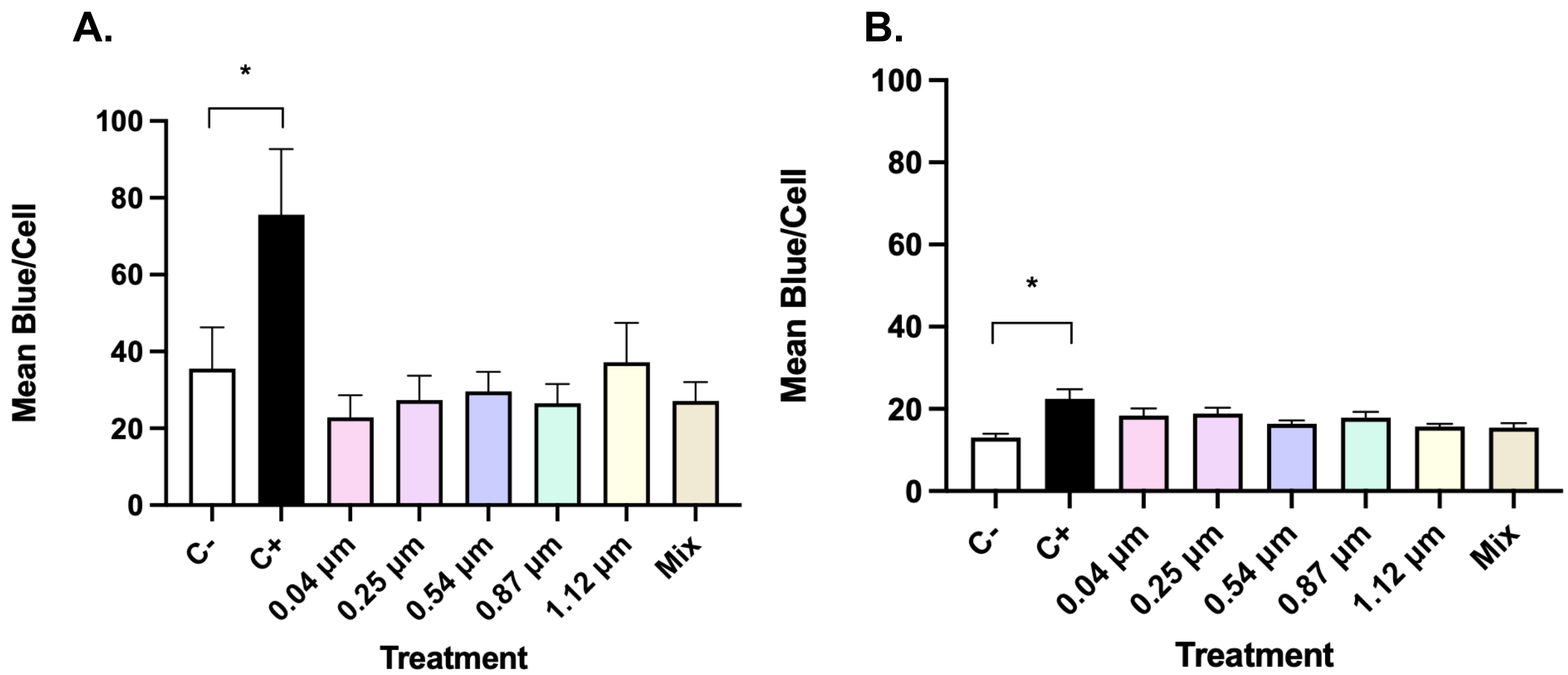

Figure S11. Assessment of plasma membrane damage in HMEC-1 and Caco-2 cells using Trypan Blue staining. HMEC-1 (**A**) and Caco-2 (**B**) cells were exposed to polystyrene nanoplastics (NPs) of 0.04, 0.25, 0.54, 0.87, and 1.12 µm for 48 h. HMEC-1 cells were exposed to 10 µg/mL NPs to minimize cytotoxicity and excessive particle burden that could compromise imaging resolution, whereas Caco-2 cells were exposed to 100 µg/mL NPs because these limitations were not observed in this cell model and to provide sufficient particle-associated signal for analysis. Following exposure, cells were stained with Trypan Blue to evaluate plasma membrane integrity. Untreated cells served as the negative control (C-), while positive control cells (C+) were exposed to Triton X-100 for 10-15 min to induce membrane disruption. Intracellular Trypan Blue staining was quantified using Gen5 software. Data were analyzed by one-way ANOVA followed by Dunnett's multiple comparisons test ($p < 0.05$).
